# Supplementary material for: Parallel Interdigitated Distributed Networks within the Individual Estimated by Intrinsic Functional Connectivity
Source: Neuron. 2017 Jul 19;95(2):457–471.e5. doi: 10.1016/j.neuron.2017.06.038 (PMC5519493; doi:10.1016/j.neuron.2017.06.038)
Supplement: Document S1. Figures S1–S8 [file mmc1.pdf]

Neuron, Volume *95*

Supplemental Information

Parallel Interdigitated Distributed Networks within the Individual Estimated by Intrinsic Functional Connectivity

Rodrigo M. Braga and Randy L. Buckner

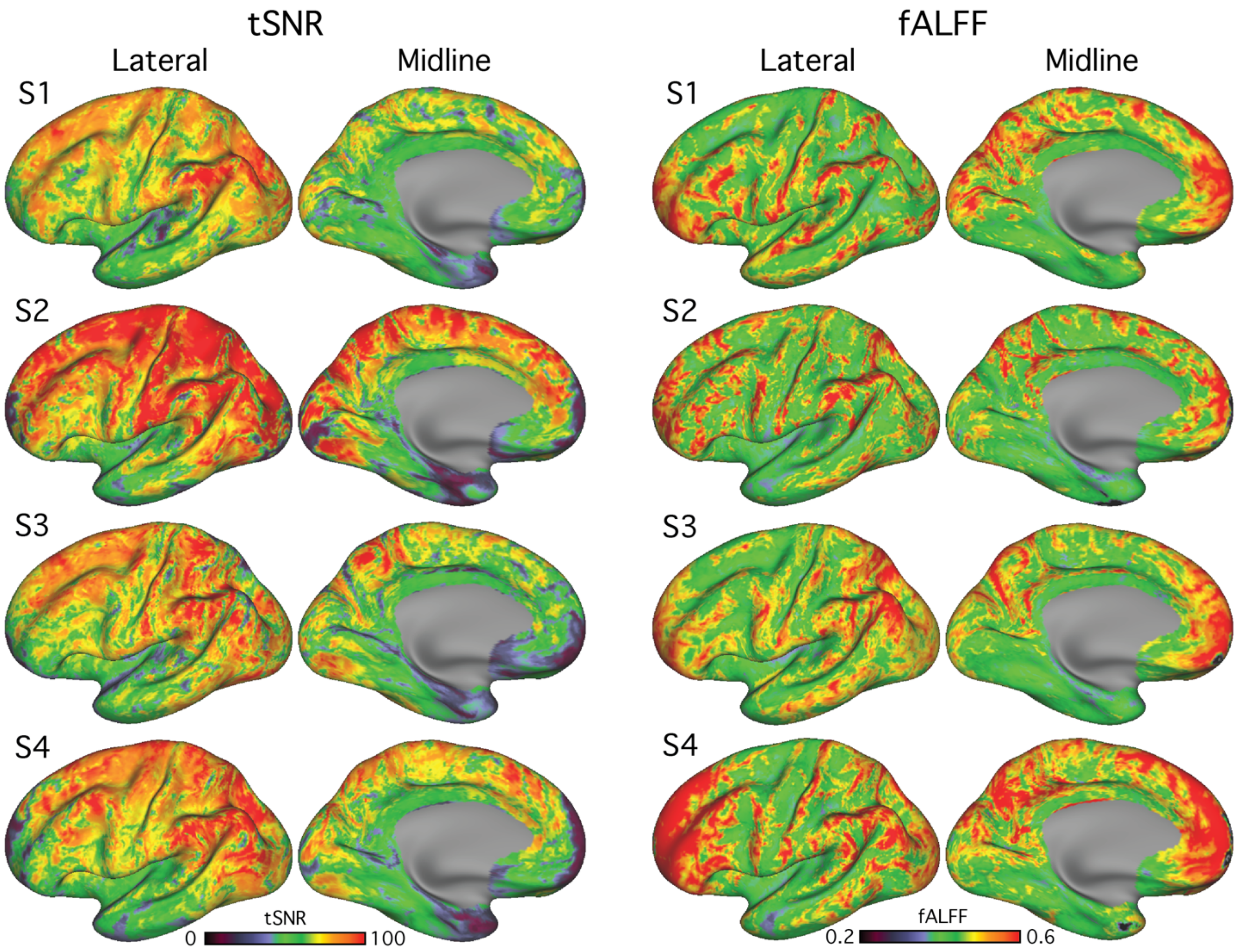

Figure S1: Estimated Temporal Signal-to-Noise Ratio (tSNR) and Fractional Amplitude of Low Frequency Fluctuations (fALFF) of the Functional MRI Data. Related to Figures 1 and 3-8 in the main text. Voxel-based tSNR and fALFF maps created from the full dataset (n = 24) for each subject (S1 – S4) are projected to the cortical surface. The maps demonstrate the spatial distribution of variance in data quality and intensity of regional spontaneous fluctuations across the cortex. While both tSNR and fALFF are decreased in regions of ventromedial prefrontal cortex and the anterior temporal lobe, there is nonetheless considerable coverage.

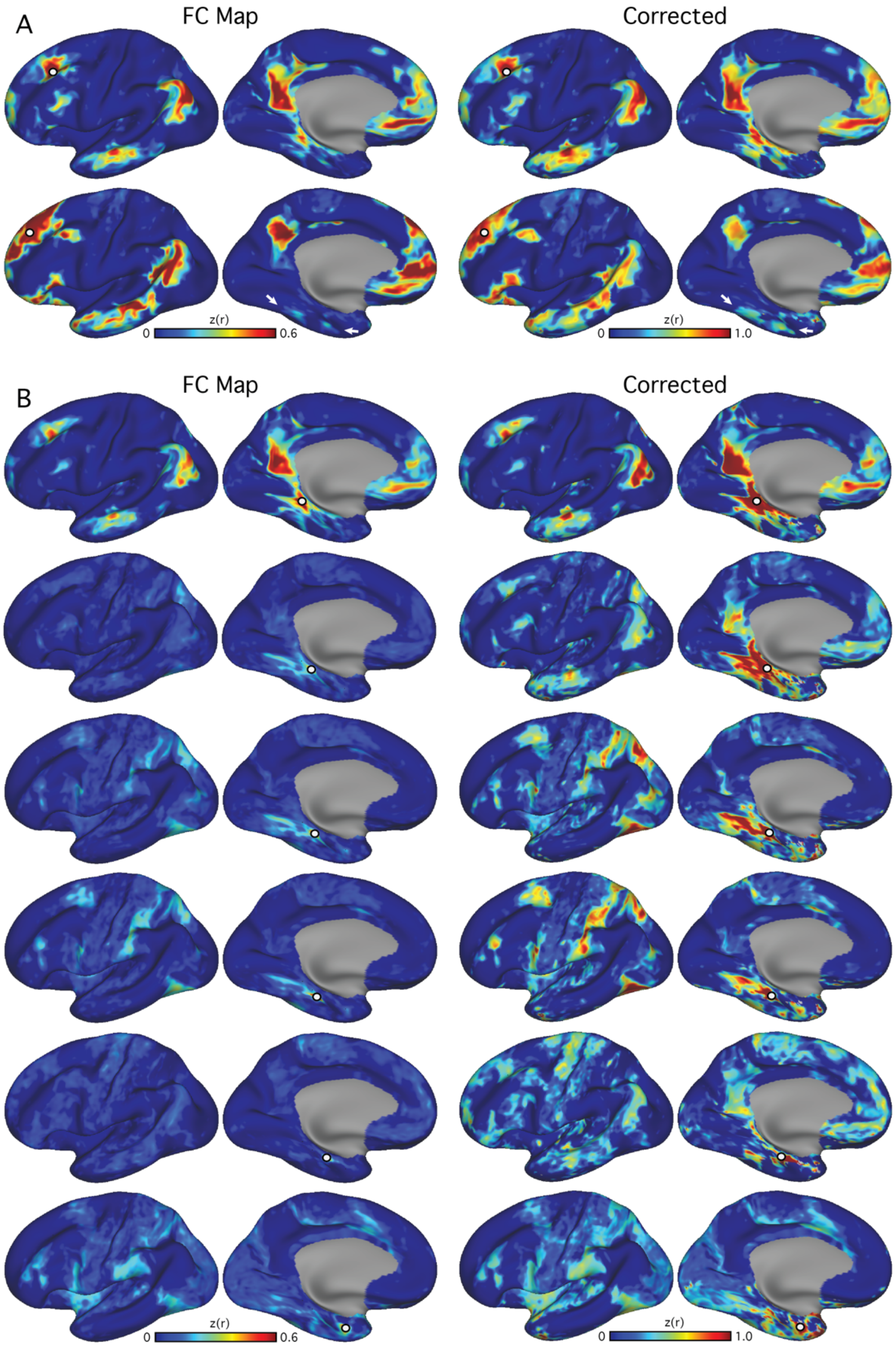
A
FC Map
Corrected
z(r)
0
0.6
z(r)
0
1.0
B
FC Map
Corrected
z(r)
0
0.6
z(r)
0
1.0

Figure S2: Additional Network Representations in Medial Temporal Lobe (MTL) Revealed by Attenuation Correction. Related to Figures 1 and 3 in the main text. The left column shows the functional connectivity maps from subject 4, normalized and averaged across all 24 scan runs, with the visualization threshold dropped to zero so that regions of low correlation strength can be observed. The right column shows the same maps after attenuation correction. A) The unthresholded connectivity maps for Default Network A (DN-A; top row) and Default Network B (DN-B; lower row) are shown, based on prefrontal seed regions. Two possible representations of DN-B are evident in the inferior temporal lobe (ITL; white arrows) at low thresholds (left column), and these representations display increased correlation values when attenuation correction is applied (right column). B) Maps display correlation for a series of seed regions moved incrementally along the MTL towards the temporal pole. Although the functional connectivity maps showed weak correlations with the rest of the cortical surface (left column), reliability correction reveals that a subregion of MTL is correlated with extrastriate visual regions possibly at or near the canonical dorsal attention network (right column). The top two rows show seed regions in parahippocampal cortex that were correlated to DN-A. The remaining rows appear to contain a mixture of signals that are equivocal as to their organization.

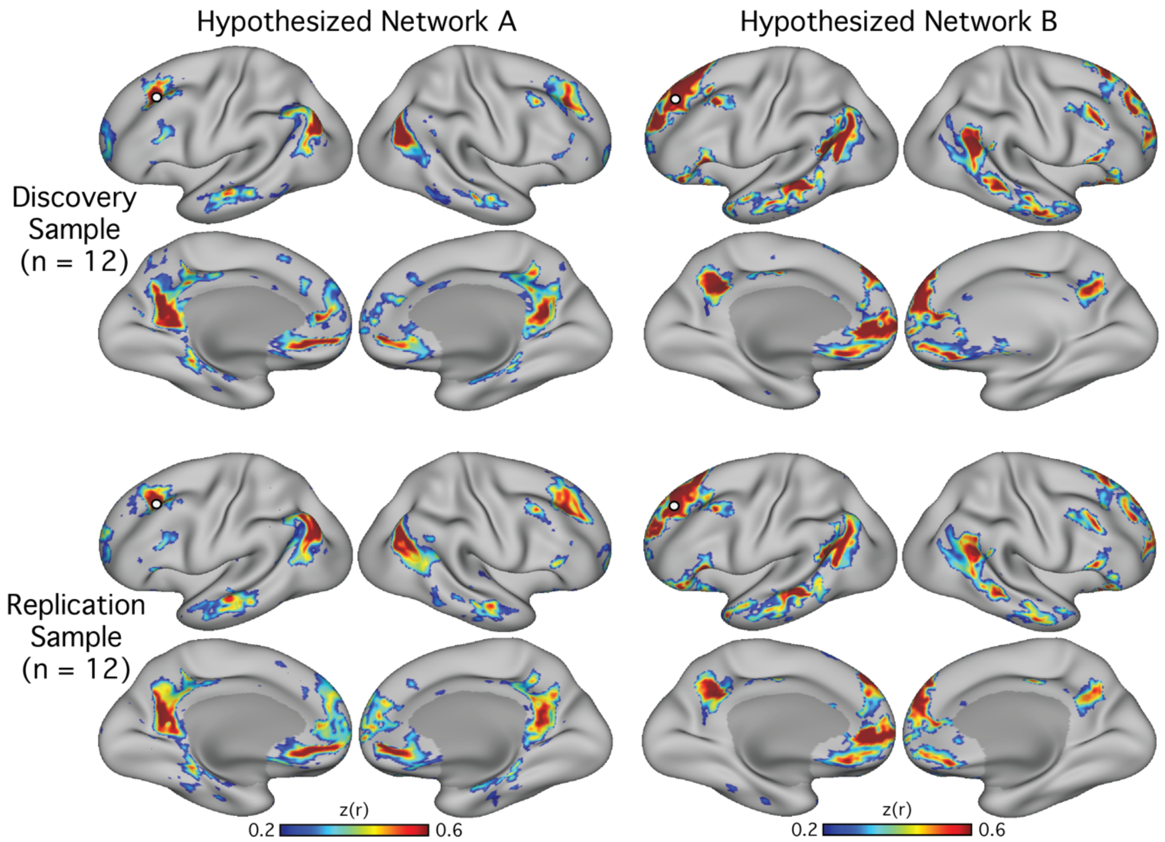

Figure S3: Spatial Details of Network Organization Replicate within the Individual. Related to Figure 1 in the main text. Illustrating the spatial stability of network organization within an individual (S1), functional connectivity maps are shown for the discovery sample (n=12 sessions, top) and the replication sample (n=12, bottom). The columns to the left represent Network A and the columns to the right represent Network B. The prefrontal seed regions are illustrated by white filled circles. In several zones, the networks are interdigitated, including the lateral temporal component of Network B which is surrounded by a component of Network A, and the medial prefrontal network components across multiple regions.

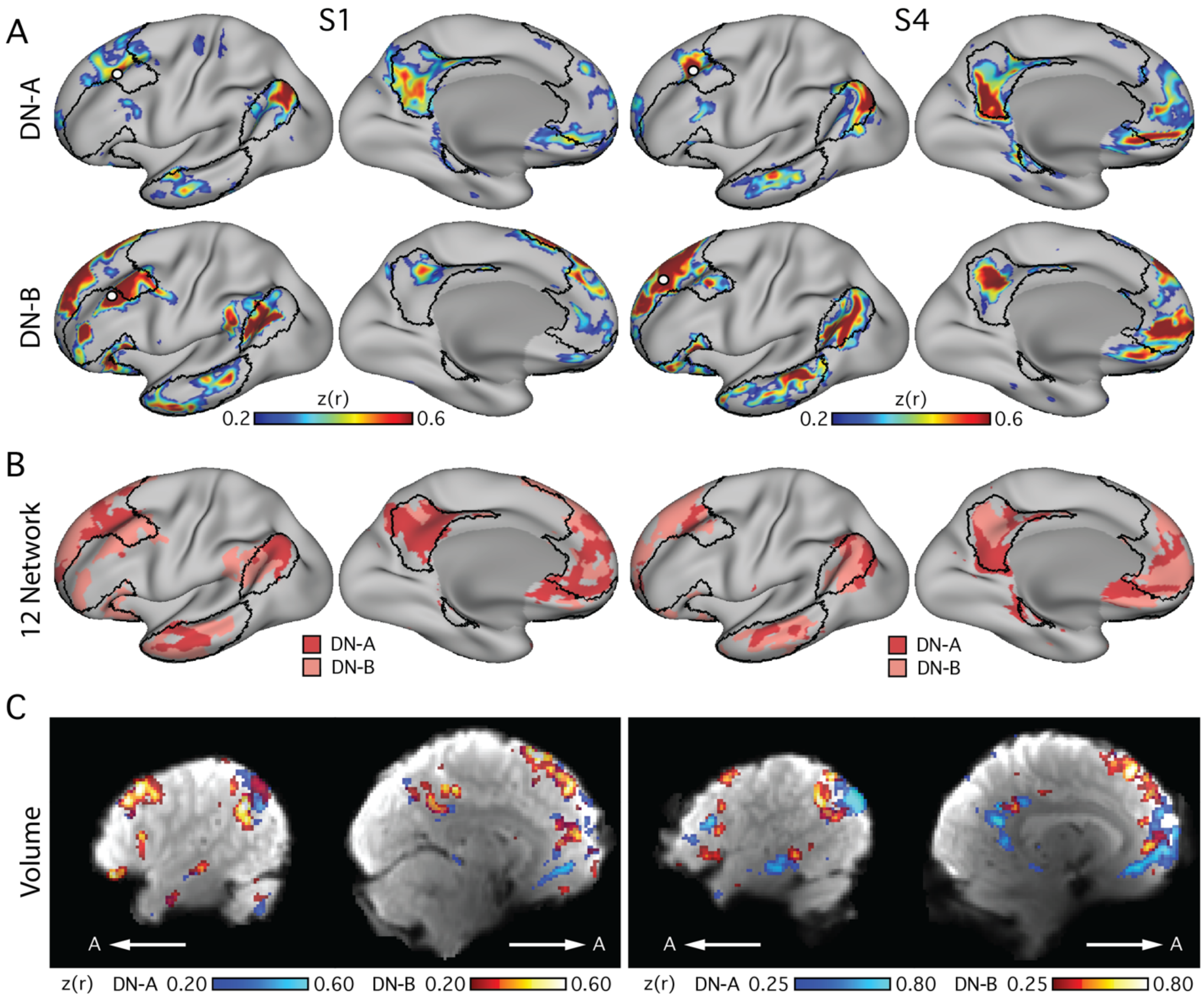

Figure S4: Parallel Interdigitated Networks Are Confirmed Using Multiple Analysis Techniques. Related to Figure 1 and 3 in the main text. Additional images confirm the observed fractionation of the default network using multiple analysis approaches (both seed-based and clustering) and anatomical visualization formats (both surface and volume). A) Seed-based, vertex-by-vertex correlation of data projected to the cortical surface, produced two connectivity maps which both resembled the canonical default network. These two distinct networks (DN-A and DN-B) are shown for subjects 1 (S1) and 4 (S4). The network border from the group-averaged default network is shown in black (taken from the 7-network parcellation from Yeo et al., 2011) to highlight how the DN-A and DN-B both contain regions that fall at or near the canonical default network. B) Networks defined using data-driven parcellation on the

surface are illustrated for each subject. For each individual, the map represents the concatenated time series from each run of the discovery dataset (n=12) clustered into 12 networks using k-means clustering. Of the 12 networks defined, the two networks at or near the canonical default network are displayed to highlight the correspondence between the clustering- and seed-based network analysis approaches. Similar networks were observed using clustering that were juxtaposed and interdigitated in temporal, parietal, medial prefrontal and lateral prefrontal cortices. C) The DN-A and DN-B networks are displayed in the volume using voxel-based functional connectivity to verify the interdigitation between networks observed on the surface is not a consequence of the cortical sampling procedure. Two separate networks are displayed that share the key features of the surface-defined networks reported in the main analysis (shown in A). The DN-A network is shown in the blue colorscale and the DN-B network is shown in the red-white colorscale, both set to a transparency level of 70%. The two networks can clearly be seen to occupy side-by-side regions throughout the brain, particularly in the temporal, parietal and medial prefrontal cortices in S4. Importantly, the distribution of representations from DN-A and DN-B in the medial prefrontal cortex shows that the two networks occupy interdigitated regions in the volume (see rightmost portions of the figures). Note that the lateral views of the brain volume are flipped to match the orientation of the cortical surfaces, such that the anterior (A and arrows) of the brain is on the left.

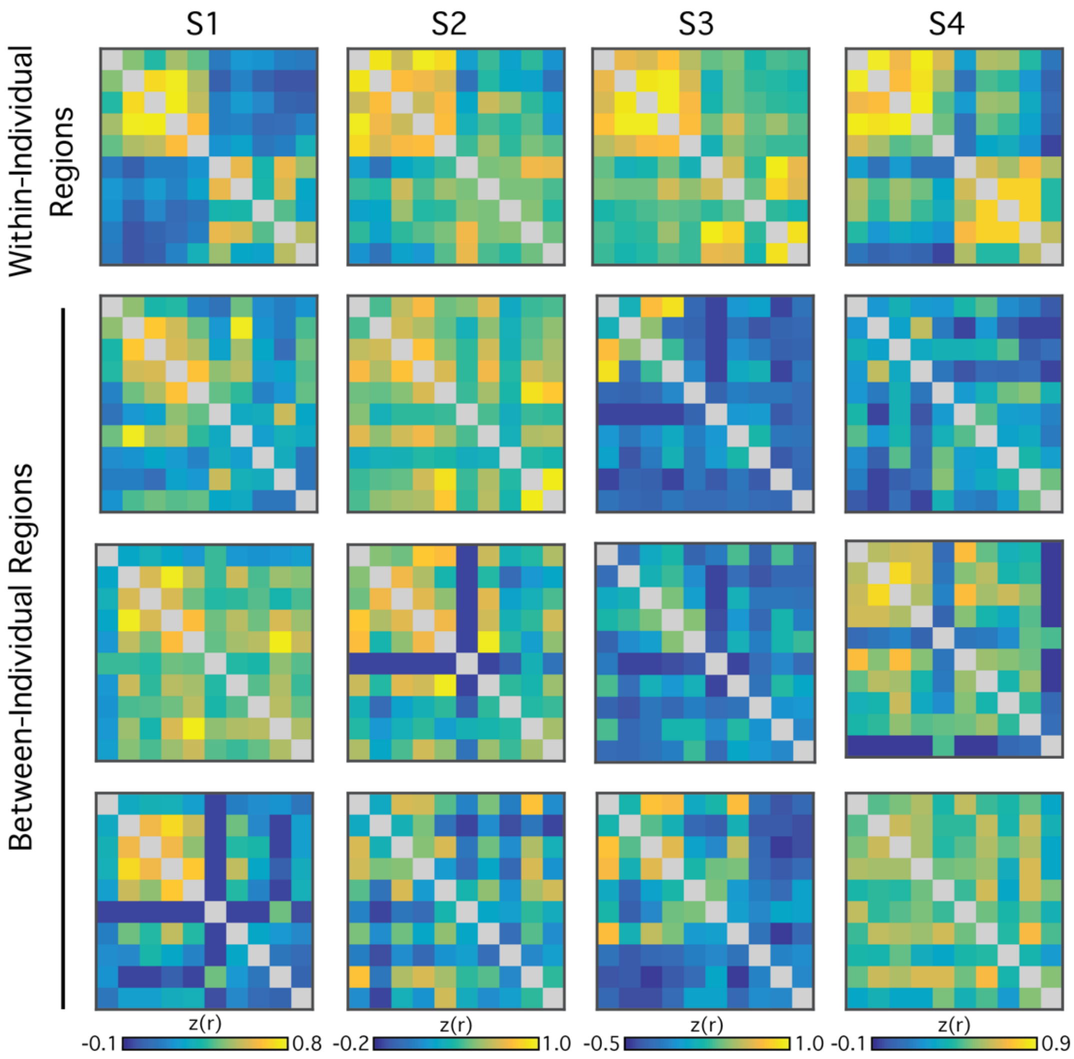

Figure S5: Network Regions Are Not Spatially Aligned Between Individuals. Related to Figure 1 in the main text. Each matrix represents the functional connectivity between the 10 paired regions comprising Default Network A and Default Network B (Figure 1). Values are the mean r-to-z transformed correlations from the 12 samples in the replication dataset. Each column represents data from one subject always using the same scale per subject (shown at the bottom). The top row presents the correlation matrix when the spatial locations of the regions are tailored to that specific subject. In each case, two clusters are evident reflecting the strong within-network correlations of the two dissociated networks (upper left quadrant and lower right quadrant in each matrix). Note further that these clusters of strong positive correlations include region pairs distributed throughout the brain that are often spatially close to regions in

the other cluster. The three rows below each within-individual matrix represent the same original data but with the matrix constructed from the seed region locations defined in the other subjects (e.g., Subject 2's regions applied to Subject 1's data). As can be seen, the clustering patterns break down and the separation of the two networks is absent in many matrices. This result illustrates that the spatial variation between individuals is enough to obscure the clear and reproducible network dissociation that is uncovered within the individuals.

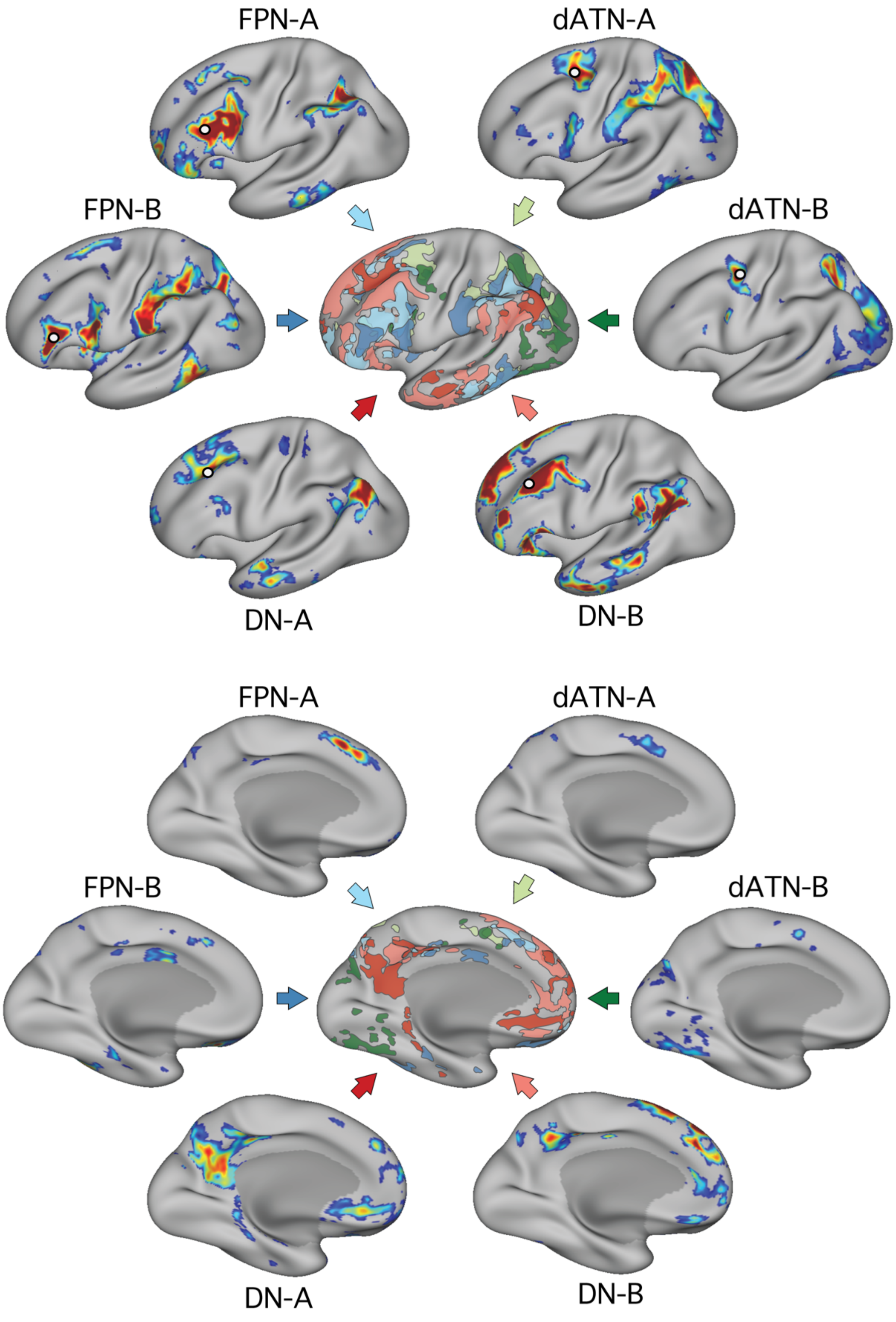

Figure S6: Diagrammatic Representation of Six Parallel Distributed Networks within Another Individual. Related to Figure 8 in the main text. The central figures show an illustration of the

six newly defined networks overlaid on the same cortical surface. The top panel shows the lateral view and the lower panel shows the medial view. The different colors correspond to the canonical network that each network resembles (Red, Default Network, DN-A and DN-B; Blue, Frontoparietal Network, FPN-A and FPN-B; Green, Dorsal Attention Network, dATN-A and dATN-B). The names of the networks are chosen based on prior literature, recognizing that the novel organization identified here may lead to a reconsideration of the functional domains. Data are from Subject 1 (see Figure 8 in the main text for Subject 4).

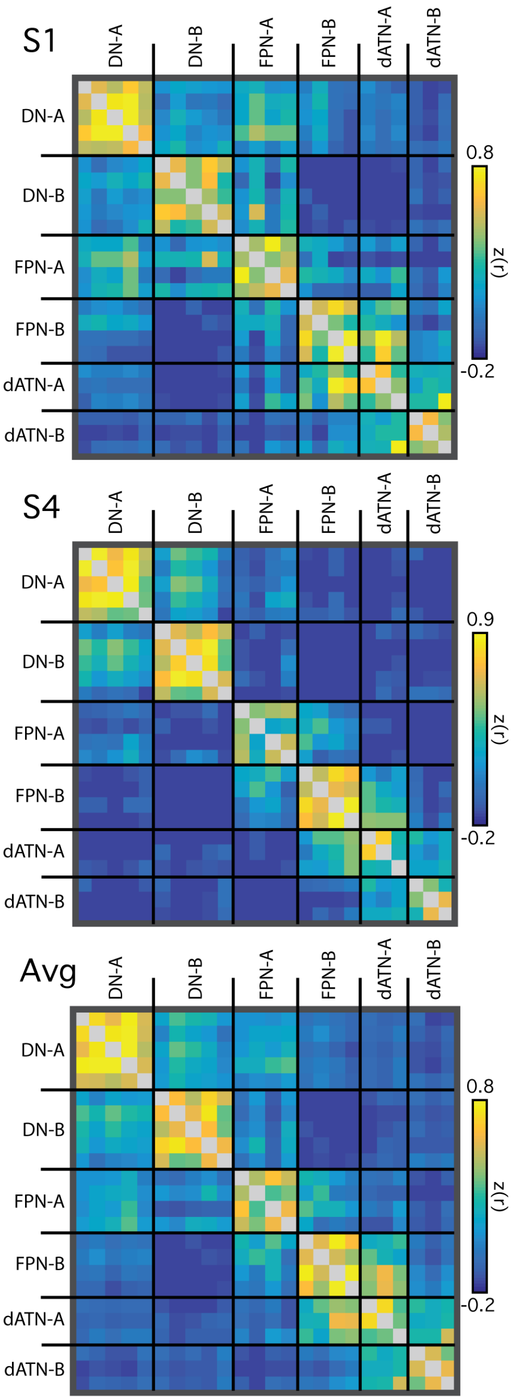

Figure S7: Correlations Between Distributed Networks. Related to Figures 4-8 in the main text. Each matrix illustrates the correlation structure from regions of the six identified networks for subjects S1 (top panel) and S4 (middle panel), and the average of the two subjects (Avg; bottom panel). Region locations are shown in Figure S8. The matrices demonstrate that there may be subtle, differential interactions between networks.

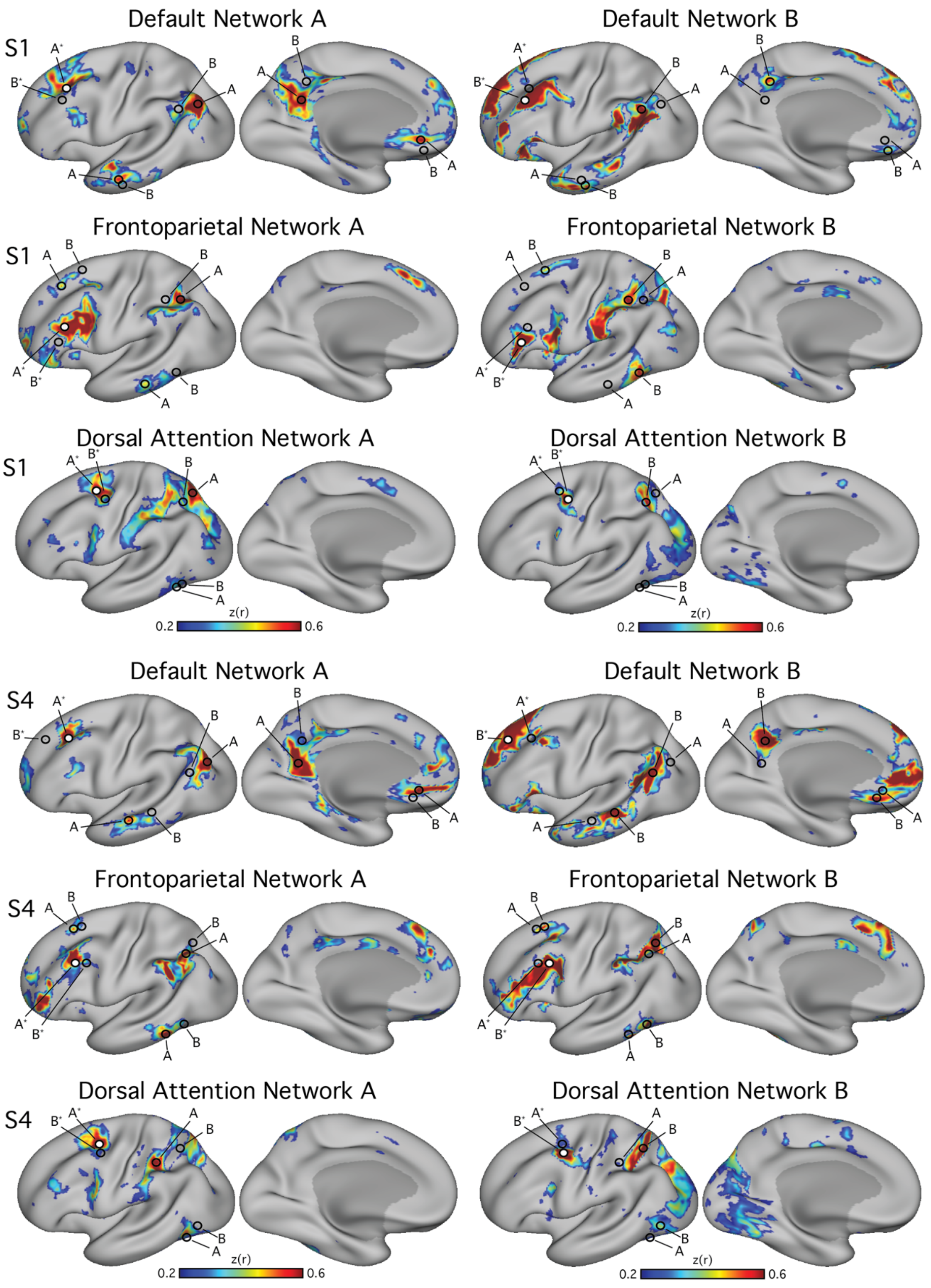

Figure S8: Network Regions for All Six Networks for Subjects 1 and 4. Related to Figures 4-8 in the main text. Panels display the correlation maps for seed regions (white filled circles) placed

in prefrontal cortex to isolate the pairs of fractionated networks within the canonical Default Network (top row for each subject), Frontoparietal Network (middle row), and the Dorsal Attention Network (bottom row). Within each row, the left column shows one network and the right column shows the parallel, spatially distinct network. The labeled regions (hollow circles) were used to estimate network correlations within- and between-networks as presented in Figure S7.
