# Supplementary material for: Parallel Interdigitated Distributed Networks within the Individual Estimated by Intrinsic Functional Connectivity
Source: Neuron. 2017 Jul 19;95(2):457–471.e5. doi: 10.1016/j.neuron.2017.06.038 (PMC5519493; doi:10.1016/j.neuron.2017.06.038)
Supplement: Document S2. Article plus Supplemental Information [file mmc2.pdf]

Neuron

Article

Parallel Interdigitated Distributed Networks within the Individual Estimated by Intrinsic Functional Connectivity

Highlights

- Within-individual characterization of brain networks reveals new spatial details
- Group-defined networks fractionate into distinct parallel networks in individuals
- Parallel networks possess closely juxtaposed regions in numerous cortical zones
- Networks share a conserved motif that may be organized along a macroscale gradient

Authors

Rodrigo M. Braga, Randy L. Buckner

Correspondence

rodrigo.braga@imperial.ac.uk (R.M.B.),
randy_buckner@harvard.edu (R.L.B.)

In Brief

Braga and Buckner examine the detailed organization of brain networks within individual people. They discovered that multiple closely juxtaposed cortical regions form parallel distributed networks. Separate large-scale networks may emerge from a common organizing principle.

CrossMark

Braga & Buckner, 2017, Neuron *95*, 457–471
July 19, 2017 © 2017 The Authors. Published by Elsevier Inc.
http://dx.doi.org/10.1016/j.neuron.2017.06.038

CellPress

CellPress

Parallel Interdigitated Distributed Networks within the Individual Estimated by Intrinsic Functional Connectivity

Rodrigo M. Braga[1,2,3,5,*] and Randy L. Buckner[1,3,4,*]
[1]Department of Psychology, Center for Brain Science, Harvard University, Cambridge, MA 02138, USA
[2]The Computational, Cognitive & Clinical Neuroimaging Laboratory, Hammersmith Hospital Campus, Imperial College London, London W12 0NN, UK
[3]Athinoula A. Martinos Center for Biomedical Imaging, Massachusetts General Hospital, Charlestown, MA 02129, USA
[4]Department of Psychiatry, Massachusetts General Hospital, Charlestown, MA 02129, USA
[5]Lead Contact
*Correspondence: rodrigo.braga@imperial.ac.uk (R.M.B.), randy_buckner@harvard.edu (R.L.B.)
http://dx.doi.org/10.1016/j.neuron.2017.06.038

SUMMARY

Certain organizational features of brain networks present in the individual are lost when central tendencies are examined in the group. Here we investigated the detailed network organization of four individuals each scanned 24 times using MRI. We discovered that the distributed network known as the default network is comprised of two separate networks possessing adjacent regions in eight or more cortical zones. A distinction between the networks is that one is coupled to the hippocampal formation while the other is not. Further exploration revealed that these two networks were juxtaposed with additional networks that themselves fractionate group-defined networks. The collective networks display a repeating spatial progression in multiple cortical zones, suggesting that they are embedded within a broad macroscale gradient. Regions contributing to the newly defined networks are spatially variable across individuals and adjacent to distinct networks, raising issues for network estimation in group-averaged data and applied endeavors, including targeted neuromodulation.

INTRODUCTION

The cerebral cortex possesses a complex tapestry of networks that interact and compete in the service of information processing. Building on a history of assigning specialized functions to brain regions, early seminal work by Norman Geschwind, Marsel Mesulam, and others proposed ideas about how distributed regions might interact to perform high-level tasks (e.g., Geschwind, 1965a, 1965b; Mesulam, 1981, 1990). A leap in progress occurred when networks began to be conceptualized within the framework of anatomical connectivity patterns in the macaque, following the availability of both retrograde (Mesulam et al., 1977) and anterograde (Cowan et al., 1972) tracers. For example, by charting the laminar pattern of anatomical projections, a hierarchical pathway emerged from areas involved in visual perception through to areas enabling motor actions (Maunsell and van Essen, 1983; Ungerleider and Desimone, 1986; Andersen et al., 1990; Boussaoud et al., 1990; see Shadlen and Newsome, 2001 for discussion). This canonical distributed network comprises primary and secondary visual areas, parietal association areas, and motor areas. These interconnected areas form a partially modular distributed network that interacts with, but is anatomically distinguishable from, other processing hierarchies (Felleman and Van Essen, 1991; Ungerleider and Desimone, 1986; Van Essen et al., 1992; see also Markov et al., 2014). Notably, this canonical network involves areas distributed across temporal, parietal, and frontal cortices. As will be illustrated below, this distributed pattern is a general motif that is apparent across multiple large-scale networks (see Goldman-Rakic, 1988).

Human neuroimaging studies are particularly useful for characterizing distributed networks because they survey the whole brain at once. Corbetta and Shulman (2002) highlighted a network currently known as the "dorsal attention network" (dATN) in the human neuroimaging literature, which is likely homologous to later-stage components of the sensory-motor hierarchy described above in the macaque (Vincent et al., 2007; Patel et al., 2015). Network analysis based on intrinsic functional connectivity (FC) consistently reveals the dATN (e.g., Fox et al., 2006; Vincent et al., 2008; see also Beckmann et al., 2005). Detailed analysis in relation to retinotopic areas recapitulates, to first approximation, the full hierarchical pathway described in the macaque (Yeo et al., 2011). Thus, while there are caveats to interpreting networks observed by FC (Buckner et al., 2013; Murphy et al., 2013; Smith et al., 2013; Power et al., 2014), the results can generate hypotheses about the organization of multiple large-scale networks that populate human association cortex.

Group-based studies using FC suggest that association cortex comprises about five major distributed networks (e.g., Yeo et al., 2011; Power et al., 2011; Doucet et al., 2011). These networks have sufficiently modular properties to be consistently identified

CrossMark

 © 2017 The Authors. Published by Elsevier Inc.
This is an open access article under the CC BY license (http://creativecommons.org/licenses/by/4.0/).

as isolated networks, even when constraints are relaxed to emphasize interactions between networks (Yeo et al., 2014). Among these, the "default network" (DN) has been extensively studied. The DN is anatomically separate from the dATN and is estimated to have expanded in hominin evolution (Buckner and Krienen, 2013; Margulies et al., 2016; see also Hill et al., 2010). Regions across the DN are correlated to a level akin to the local sensory and motor networks despite being widely distributed (Power et al., 2011; see also Greicius et al., 2003). Tracer studies suggest that the DN discovered in the human may have an anatomically connected homolog in the macaque (Buckner et al., 2008; Binder et al., 2009; see also Margulies et al., 2009; Buckner and Krienen, 2013). Several other networks, near the dATN and DN, each have their own distributed organization (Yeo et al., 2011; Power et al., 2011; Doucet et al., 2011).

Several principles emerge through examining relations among multiple large-scale networks. First, the distributed networks all have an organization that is similar to the dATN and DN, with each network possessing frontal, temporal, posterior parietal, frontal midline, and posterior midline components. Depending on how the network is estimated, certain components can be underemphasized, e.g., the dATN is sometimes described without a frontal midline component, especially when "winner-take-all" network assignments are used (e.g., Yeo et al., 2011). Targeted analyses often reveal there is a midline component (e.g., Figure 5 in Fox et al., 2006; Figure 32 in Yeo et al., 2011). The distributed networks follow a general motif that is *roughly* conserved even though each network contains spatially distinct regions. Power et al. (2011) further noted that the networks have similar spatial arrangements at their interfaces in multiple zones of cortex (e.g., temporal, parietal, frontal). That is, if a network lies side by side with another network in parietal cortex, it is also likely to do so in frontal cortex. This echoes features proposed by Goldman-Rakic (1988) (her Figure 4) and Mesulam (1981) (his Figure 4) based on anatomical data. Second, while the broad architecture follows a general motif, there are differences that distinguish the networks. For example, while the dATN is coupled to visual and motor regions, the DN is instead coupled to the hippocampal formation, perhaps reflecting a mnemonic functional anchor (Greicius et al., 2004; Vincent et al., 2006; Buckner et al., 2008; Andrews-Hanna et al., 2010).

The discovery that cerebral association cortex possesses multiple distributed networks is a major milestone for the field. However, reliance on group-averaged estimates raises questions. A first open question concerns whether the present convergence on major networks reflects the correct level of description. Similarities between human neuroimaging and monkey anatomical findings suggest that the major networks described to date capture true organizational features. Nonetheless, several reports note distinctions that fractionate the larger major networks, either locally within a region or in complex ways across distributed regions. For example, Smith et al. (2013) presented a clustering analysis that illustrated both high-level structure that recapitulated the major networks and a substructure that included spatial distinctions between nearby regions of cortex (see also Smith et al., 2012). In the study by Yeo et al. (2011), they showed that the major networks broke down further when more fine-grained network structure was examined (their 17-network parcellation). Power et al. (2011) noted at least two network subcomponents ("subgraphs" within their framework) that were not captured in the major network descriptions. In detailed analysis of the DN, there have been several proposed schemes to delineate subnetworks (e.g., Andrews-Hanna et al., 2010, 2014; Leech et al., 2011, 2012; Braga and Leech, 2015). We do not attempt to integrate these various findings here but point out that analyses of group data have always possessed features that are not fully accommodated by assuming a small number of major networks.

A second open question is whether there are global properties of organization that span across the networks. Margulies et al. (2016) recently proposed a macroscale organization of networks that moves outward from sensory-motor networks on one end to the DN on the other end. This echoes an idea advanced by Mesulam (1998) that the cortex exhibits a hierarchical organization progressing from unimodal areas to integrative transmodal areas (see also Jones and Powell, 1970). The repeating motif and spatial juxtaposition of multiple networks suggest that the macroscale organization of the distributed networks might be partially explained by developmental anchors or gradients (Buckner and Krienen, 2013; Margulies et al., 2016; Krienen and Buckner, 2017). Detailed analysis of cortical network architecture may provide evidence for or against macroscale gradients that span networks.

To make progress on these questions, we need to move from group-level description to the finer spatial scale that is accessible when studying organization within the individual. The majority of the literature on network architecture is based on averaging data from groups of spatially normalized individuals. Group averaging was necessary in positron emission tomography (PET) studies as radiation places severe restrictions on repeat imaging. A major technical breakthrough came with the ability to average brain volumes across individuals to boost signal to noise (e.g., Fox et al., 1985; Evans et al., 1992; Friston et al., 1995). However, group-averaging approaches are limited due to blurring over anatomical and functional variability (Steinmetz and Seitz, 1991; Silbersweig et al., 1993).

Anatomical variability refers to the complex geometry of the brain's gross structural morphology (e.g., sulci and gyri) that must be normalized across subjects. Surface-based normalization has improved this technical hurdle for the cerebral cortex (Fischl et al., 1999; Van Essen, 2005; Robinson et al., 2014), but misregistration is still a challenge. Functional variability refers to the spatial arrangement of functional zones on the cortical surface. Functional organization is likely derived from microscopic anatomical features, such as areal organization or anatomical subdomains that possess distinct connections. The point here is that these local organizational properties are not fully accounted for by gross anatomical differences between individuals. Histological data have illustrated that the border locations, size, and shape of architectonic areas vary on the surface between individuals (e.g., Rademacher et al., 1993; Rajkowska and Goldman-Rakic, 1995; Amunts et al., 1999, 2000; Caspers et al., 2006; Fischl et al., 2008; Henssen et al., 2016). Thus, even if individuals could be brought into complete anatomical alignment, spatial blurring across architectonically defined areas would persist. An additional consideration is that cortical organization may possess complex topography that has side-by-side

juxtapositions and finely interleaved structure. Fine spatial arrangements may reflect the complex geometry of small cortical domains (e.g., Gordon et al., 2017a; Glasser et al., 2016), such as has been hypothesized for face patches (Moeller et al., 2008), or the fine-grained structure due to topographically arranged projections such as is present for eccentricity in the early visual system (e.g., Maunsell and van Essen, 1983). Normalization across subjects may blur these spatial features.

Recent human neuroimaging studies focusing on individuals have noted fine spatial details that are lost or attenuated by group averaging. Fedorenko et al. (2012) demonstrated that a language-preferential region in prefrontal cortex (PFC) appears "as an island" between regions showing domain-general response properties. The exact positioning of the island moves from person to person. Michalka et al. (2015) characterized interdigitated regions of PFC belonging to separate auditory and visual networks whose exact positions vary considerably across subjects (Figure S1 in Michalka et al., 2015). Laumann et al. (2015) recently highlighted a network implication of closely juxtaposed small regions. Two distinct networks were found in an individual using nearby seed regions in left PFC; however, when the same two regions were applied to group-averaged data, a single distributed network emerged (Figure 7 in Laumann et al., 2015). In an analysis motivated by architectonic subdivisions of nearby prefrontal areas BA 44 and BA 45, Jakobsen et al. (2016) (their Figure 11) observed a similar separation of networks in the individual. Glasser et al. (2016) noted that whereas in the majority of cases a single contiguous region of lateral PFC was found to belong to a left-lateralized language network (Figure S8 in Glasser et al., 2016), a minority of individuals displayed two "split" non-contiguous regions. In a comprehensive analysis of individual-specific network features, Gordon et al. (2017b) identified numerous reliable features that were not captured in group estimates of network organization. These features form a distributed set of patches across the cortical mantle that were "too infrequently present and/or spatially variable relative to their size to emerge in group-average data." Thus, analysis of individual brains reveals regional and network features that are underemphasized in group-averaged analyses.

Motivated by these findings, we conducted an extensive set of analyses focused on the individual. We discovered that the DN could be reliably subdivided into parallel networks within the individual. Similar separations were made for other major networks. Regions of the separate networks lay side by side across distributed zones of cortex and were sufficiently variable between individuals to obscure their existence in group-averaged analyses. To make these observations, we analyzed data from four individuals scanned repeatedly over 24 sessions.

RESULTS

High Signal-to-Noise and Full-Brain Coverage Was Achieved in Each of Four Individuals

The present study acquired extensive data over many functional MRI (fMRI) sessions in the same individuals. Two estimates of data quality, slice-based temporal signal-to-noise ratio (tSNR) and fractional amplitude of low-frequency fluctuations (fALFF; Zou et al., 2008), were calculated for each participant. Across each subject's 24 scans, slice-based tSNR ranged between 127.2 and 336.3 for S1, 264.0 and 461.2 for S2, 123.2 and 286.6 for S3, and 204.7 and 493.1 for S4. Absolute motion (i.e., the accumulated displacement in each run) ranged between 0.303 and 1.505 mm for S1, 0.209 and 1.181 mm for S2, 0.474 and 1.651 mm for S3, and 0.267 and 1.765 mm for S4. Spatial properties of tSNR and fALFF are affected by susceptibility artifacts (e.g., Ojemann et al., 1997). Figure S1 displays voxel-based tSNR and fALFF maps projected onto the cortical surface. High voxel-based tSNR and fALFF were achieved across nearly the entire cortical mantle, including ventral PFC and portions of the anterior and ventral temporal lobe.

Distinct Distributed Networks Fractionate the Canonical Default Network within the Individual

The goal of the present study was to identify spatially detailed features of network organization in the individual. For each participant, half the data were used for discovery (n = 12) of network features that were later tested in the remaining independently collected sessions (n = 12). The discovery datasets were analyzed blind to the hypothesis-testing datasets. An interactive seed selection and FC map viewing platform was established using a bespoke high-resolution cortical mesh so as to minimize spatial blurring during interpolation and allow networks to be defined with high precision by selecting individual vertices.

In the discovery data, the observation was made in one subject that two similar, but distinct, networks could be observed from two seed vertices selected from adjacent regions of left lateral PFC (Figure 1). Both networks were distributed across inferior parietal, lateral temporal, medial PFC, and posteromedial cortices and resembled the canonical DN but with distinct nodes. The two networks had components that were closely neighboring but separate in numerous cortical zones, including temporal and ventral medial PFC, suggesting that the DN may be comprised of distinct neighboring networks. We refer to these hypothesized networks as Network A and Network B (Figure 1). Investigating additional participants yielded a similar distinction.

An important difference between the networks was that Network A showed correlation with a region in parahippocampal cortex (PHC), whereas no such evidence was found in Network B, even when additional and more sensitive analyses were carried out focused on this region (Figure S2). The presence of correlation with medial temporal lobe structures is anticipated (e.g., Greicius et al., 2004; Vincent et al., 2006). However, that the coupling was to one hypothesized network and not the other was unexpected.

The recurrence of the distributed pattern of regions across the four participants provided strong evidence for two dissociable networks (Figure 1).

Double Dissociation of the Two Networks within the Individual

The independent replication data were used to formally test the hypothesis that Network A was dissociable from Network B. Using only the discovery datasets, a priori regions (single vertices) were selected that maximized the separation of the networks from the two lateral PFC seed regions in the main regional zones of the cortex (temporal, inferior parietal, posteromedial,

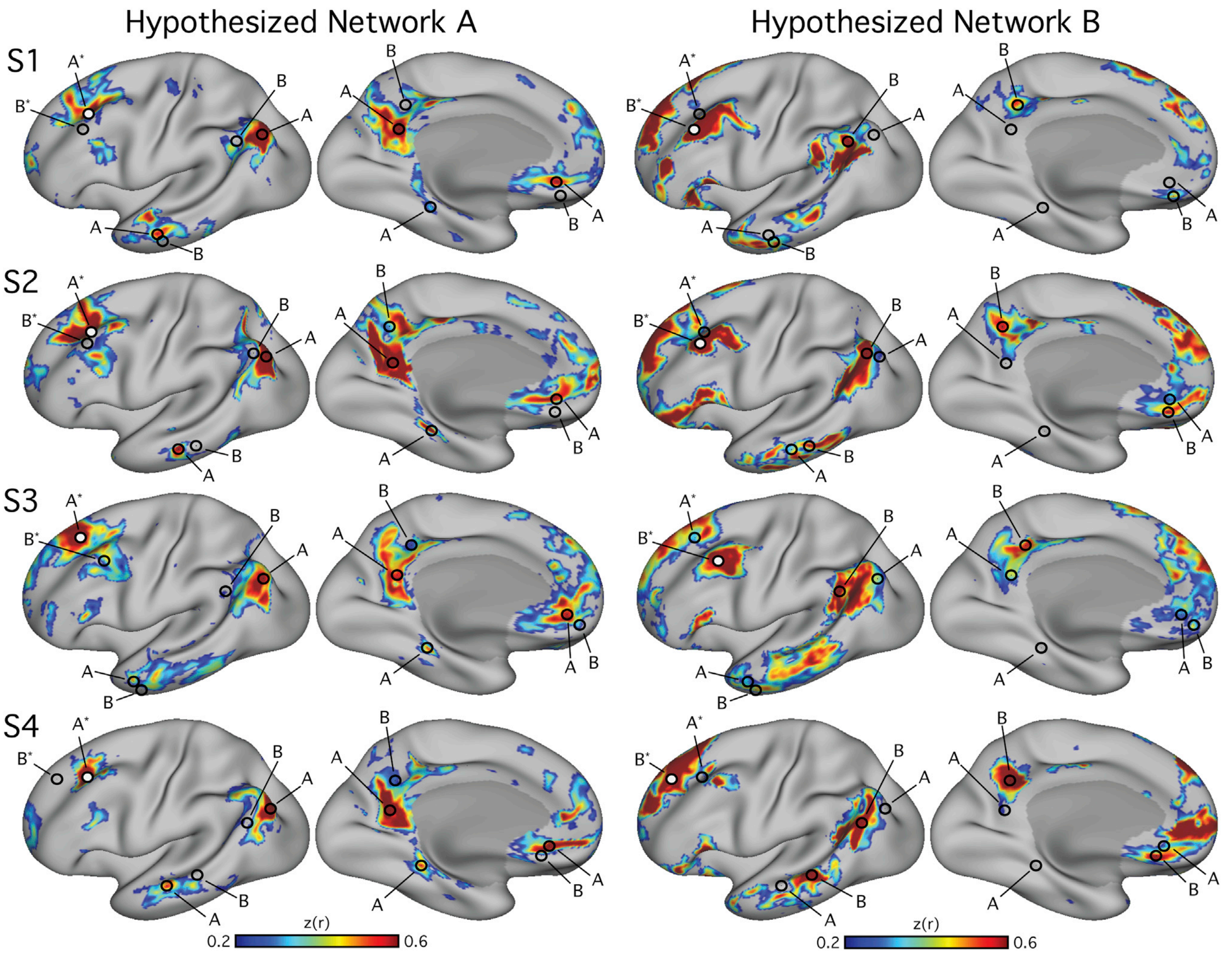

Figure 1. Two Parallel Interdigitated Distributed Networks at or near the Canonical Default Network Are Revealed by Functional Connectivity within Individuals

Each row illustrates functional connectivity (FC) maps from a single subject (S1–S4). Two networks were observed in each individual. Subject-specific seed regions were placed in the left lateral PFC of the discovery dataset (white filled-in circle). The seed region labeled A* yielded Hypothesized Network A (left) and the seed region labeled B* yielded Hypothesized Network B (right). Note that throughout the cortex, Networks A and B are adjacent to one another with slightly varied positions from individual to individual. Hypothesized Network A includes posterior inferior parietal lobule, lateral temporal cortex, ventromedial PFC, retrosplenial/ventral posteromedial cortex, and parahippocampal cortex. Hypothesized Network B includes the temporoparietal junction, lateral temporal cortex, an inferior region of ventromedial PFC, a dorsal region of anteromedial PFC, and posterior cingulate cortex. Regions (hollow circles A and B) were selected to formally test the distinction between the two networks in independent data. The surfaces are rotated by 19° along the y plane to better show the ventromedial PFC and intraparietal sulcus. The same views are used in accompanying figures.

and medial PFC) and a region in PHC. These a priori regions were targeted within each subject to locations where contiguous vertices could be observed in Network A and Network B (Figure 1). Using the independent replication dataset ($n = 12$ in each subject), time courses were extracted from each subject's a priori regions. The r-to-z transformed Pearson's product moment correlation was computed between the two lateral PFC seed regions and each distributed test region (Figure 2). Two-way ANOVA was used to test the dissociation between the two PFC seed regions and the regions in each cortical zone. The critical test was whether there would be significant interactions between seed and target regions belonging to Networks A and B. The presence of interactions across all distributed zones of the cortex would provide strong evidence that there was a network dissociation across the cortex.

Critically, the multiple tests across cortical zones and subjects targeted convergent evidence for network dissociation. Twenty 2 × 2 ANOVAs were carried out across four subjects and five cortical zones (temporal, inferior parietal, posteromedial, medial PFC, and PHC). The PHC region from Network A was paired with the posteromedial region from Network B. All 20 ANOVAs were individually significant ($p < 0.01$) with most showing crossover interactions (Figure 2). One exception was the medial PFC interaction that, while significant in all four subjects,

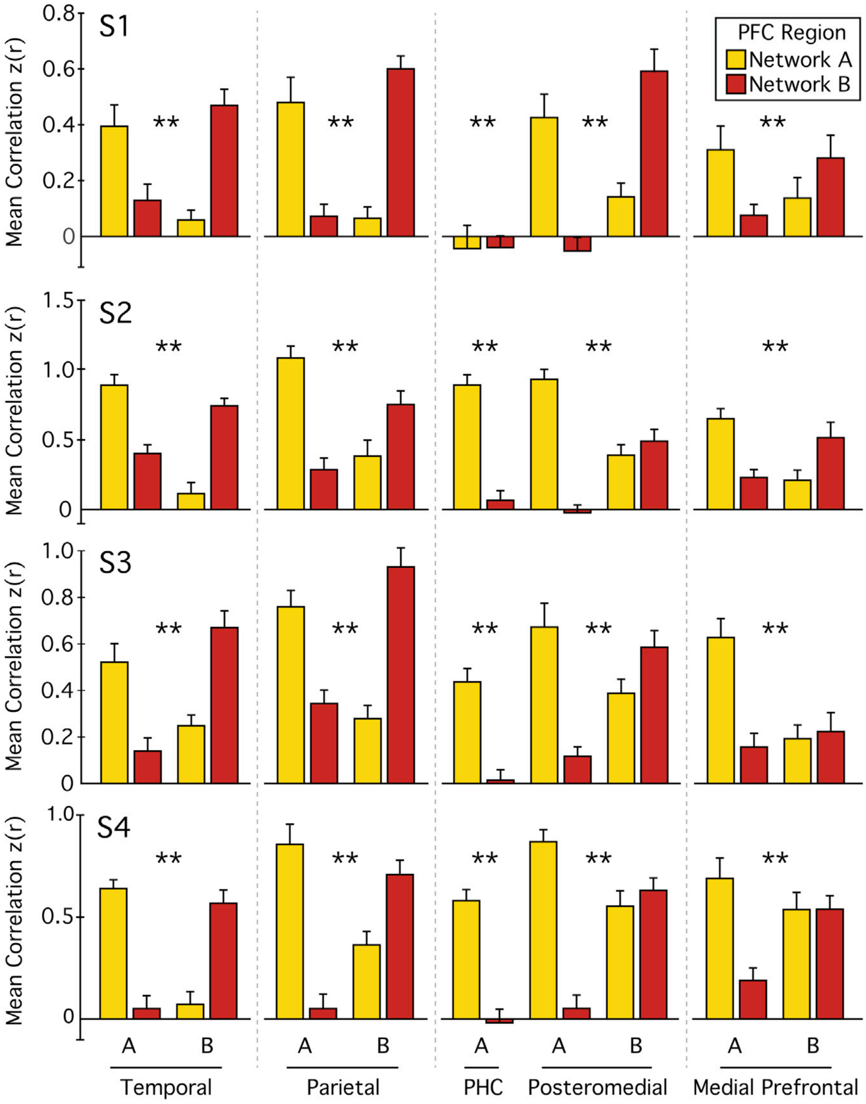

Figure 2. Parallel Distributed Networks Are Statistically Dissociated Using Independent Data

Functional correlation strength was computed between the two PFC seed regions yielding Network A and Network B and the pairs of adjacent seed regions in lateral temporal (Temporal), inferior parietal (Parietal), Posteromedial, and Medial PFC cortices (regions shown in Figure 1). This yielded a 2 × 2 contrast for each zone of cortex (e.g., Networks A and B's PFC regions against Networks A and B's Temporal regions). An additional seed region in PHC was grouped with Network B's posteromedial region. Correlations with Network A's PFC region are shown in yellow and Network B's in red. Bars represent the mean from the 12 sessions of the hypothesis-testing dataset with SEM. All 20 2 × 2 ANOVA tests were significant (**$p < 0.01$), with most showing a cross-over interaction.

demonstrated a clear crossover in two subjects and minimal difference for the most ventral region in two subjects. Another exception was the PHC interaction, which did not show a clear crossover interaction in S1. Note that, in most cases, the crossover interactions are present even where regions are extremely close to one another.

While the above analyses formally test the double dissociation, another source of evidence is that the spatial patterns replicate within individuals across discovery and replication (hypothesis-testing) datasets (Figure S3). Additional analyses examined alternative methods for identifying these separate networks, including using data-driven clustering and estimation of connectivity patterns in the volume in addition to the surface (Figure S4). Visualization in the native volume is important because projection to the cortical surface can induce fractionations of single regions into multiple regions if they fall near sulcal boundaries. The combined results illustrate a robust double dissociation between two distributed networks that fractionate the canonical DN across numerous cortical zones (Figure 3).

The Importance of Examining Network Organization within the Individual

The organization of the dissociated networks suggests why they might evade group-averaged analyses. Spatial "hurdles" to group averaging have been previously reported (e.g., Fedorenko et al., 2012's Figure 1; Laumann et al., 2015's Figure 7). To quantify the effect of spatial misalignment between individuals, we took an approach using spatial yoking between the individuals. For each individual, her spatially optimized regions from the discovery dataset were applied to her independent replication data, yielding an unbiased correlation matrix (Figure S5). Two clear clusters of strong within-network and minimal between-network correlations were observed in each individual, with S1 and S4 showing the strongest patterns. This illustrates that widely distributed regions can show strong correlation with one another, while spatially adjacent regions can be embedded in distinct correlated clusters. The same correlation matrices showed minimal structure when regions from one person were applied to another (e.g., Subject 1's regions were used to generate a matrix using Subject 2's fMRI data), highlighting the importance of respecting the exact spatial details present within an individual.

Topography of Multiple Distinct Networks within the Individual

The above analyses establish evidence for two distinct neighboring networks that are likely components of what has been studied as the DN. This unexpected observation prompted

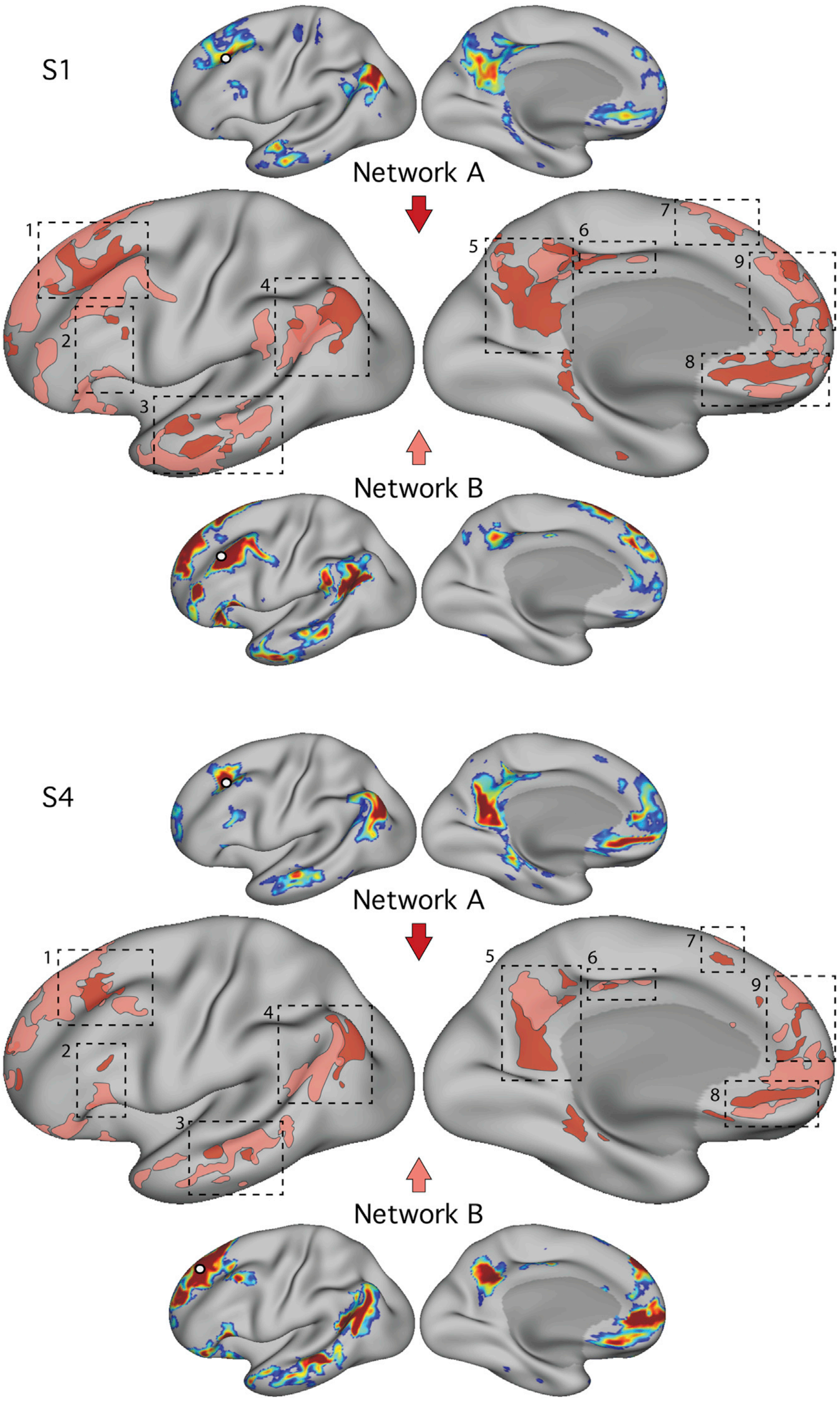

Figure 3. Parallel Distributed Networks Contain Juxtaposed Regions in Numerous Cortical Zones

The two dissociated networks near the canonical default network, Network A and Network B, are shown for two subjects (S1 and S4) in a schematic form on the same cortical surface representation. The dashed boxes highlight nine cortical zones where neighboring representations of the two networks were found including: (1) dorsolateral PFC, (2) inferior PFC, (3) lateral temporal cortex, (4) inferior parietal lobule extending into the temporoparietal junction, (5) posteromedial cortex, (6) midcingulate cortex, (7) dorsomedial PFC, (8) ventromedial PFC, and (9) anteromedial PFC. Some zones, including the dorsal region along the PFC (labeled 7), are subtle, but consistent, in all subjects, suggesting that there exists small, closely juxtaposed components of the two dissociated networks.

us to explore how these two networks relate to additional large-scale networks, the frontoparietal control network (FPN) and the dATN. Two questions drove these analyses. First, are the other large-scale networks themselves fractionated and, if so, how are the newly detected networks organized? Second, taken as a group, do the multiple large-scale networks possess consistent spatial relations between networks?

For these analyses, all 24 data sessions were combined to provide best estimate maps for each participant. Seed regions were placed in the left lateral PFC and the frontal eye fields to identify the FPN and dATN, respectively. For the FPN, two seed regions were selected that revealed networks that maximized the following features: (1) the networks contained strong representations in intraparietal, inferior temporal, and dorsomedial PFC, and (2) the networks occupied neighboring, but distinct, regions in each cortical location. For the dATN, similar criteria were applied, but the distribution instead included superior parietal, occipitoparietal, and occipitotemporal components.

As with the DN, both the FPN and dATN were fractionated into two distinct, parallel networks within the individual, identifying a total of six networks with close spatial arrangements (Figure 4). The effects were clearest in S1 and S4 and suggestive in the other subjects. In each case, the networks both resembled the canonical group-average network but inhabited separate subregions (Figure 5).

Important organizational details were evident when all six networks were visualized simultaneously. Figures 6 and 7 focus on the anterior midline and parietal and temporal lobes to illustrate two organizational features. First, regions from each network are often located in distinct locations in each cortical zone. The white lines in Figures 6 and 7 serve as landmarks to highlight non-overlapping features of the networks. Second, the networks display a fine-scale interdigitation. For example, in the anterior midline,

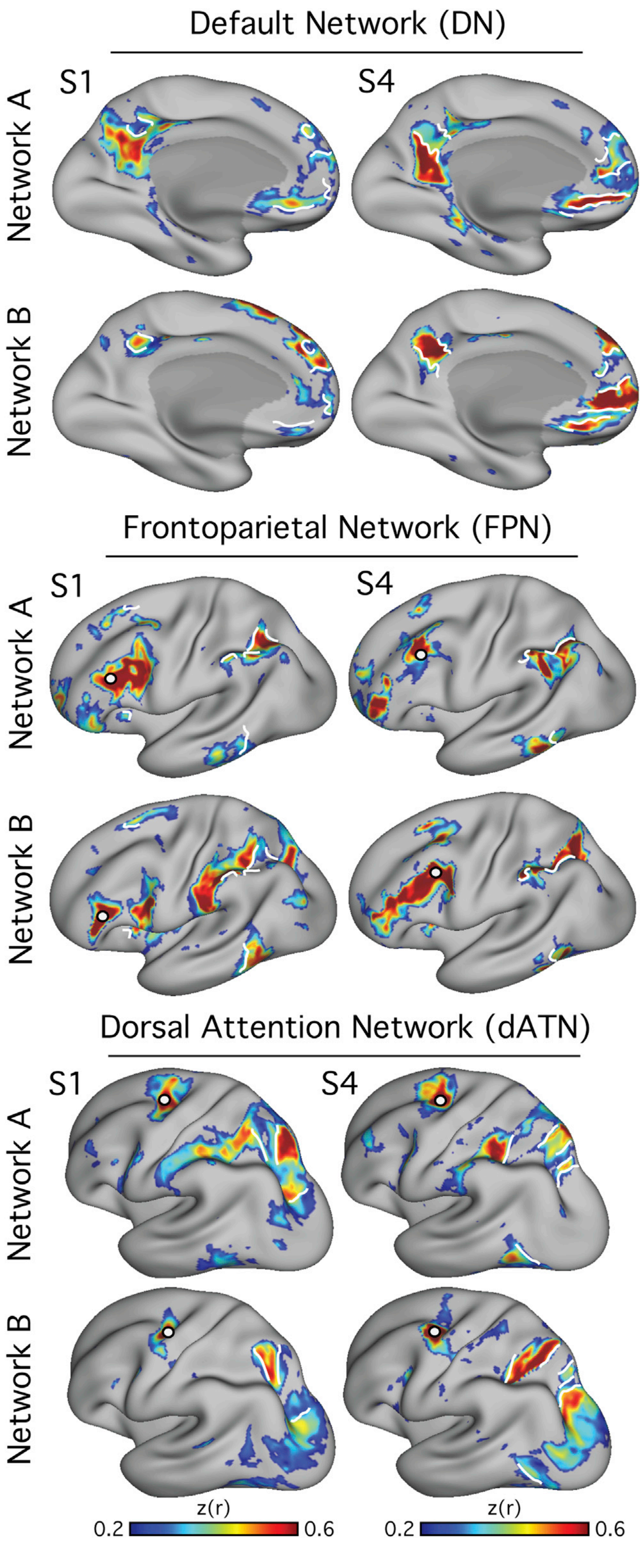

Figure 4. Multiple Parallel Interdigitated Distributed Networks at or near the Canonical Frontoparietal Control and Dorsal Attention Networks Estimated by Functional Connectivity within Individuals

Best estimate maps (using all 24 sessions in each individual) of Networks A and B that fractionate the default network are illustrated (top). Maps from two subjects (S1 and S4) are displayed. The canonical Frontoparietal Network (middle) and Dorsal Attention Network (bottom) also each fractionate into two juxtaposed networks. Seed regions are illustrated by filled white circles.

a broad ventral to dorsal progression is observed (Figure 6); however, particularly for the DN, the representations from Network B were positioned in between representations from Network A. Similarly in the temporal lobe, the representation from Network A is largely surrounded by representations from Network B (Figure 7). In the parietal lobe, a broad posteroventral to anterodorsal progression is observed across the networks, with each of the six networks inhabiting a distinct region (Figure 6). The fractionated Networks A and B of the canonical dATN showed three or more separate regions, as visualized on the surface, which sequentially alternated along the intraparietal arc of the canonical dATN (Figures 4, 5, and 6).

Figures 8 and S6 show a diagrammatic representation of the six different networks in two subjects to highlight the close, parallel nature of their organization within frontal, parietal, and temporal lobes. For an additional analysis, which should be considered descriptive, a correlation matrix was constructed (Figure S7) using regions chosen from all six networks (Figure S8). Data from all 24 sessions were used to construct each matrix. Given that the regions were defined and tested on the same data, the specific quantitative values of the within-network correlations should not be interpreted; however, the between-network correlations reflect an unbiased estimate of interactions between networks. Of interest, certain networks showed hints of interactions with other networks. For example, the FPN-A showed slightly elevated correlation with DN-A; the FPN-B showed slightly elevated correlation with dATN-A. These suggestive interactions may be due to spatial blurring or to biologically meaningful factors and are presented purely for their ability to generate future hypotheses.

DISCUSSION

The present study examined the organization of large-scale distributed networks within the individual. We discovered that the canonical DN fractionates into two parallel networks that have juxtaposed regions throughout the cerebral cortex. Motivated by this discovery, we examined the dATN and FPN and found that each of these canonical networks also fractionates into two parallel networks. The organization of the six identified networks was charted and found to have a spatial progression in multiple zones of cortex. These results are consistent with the ideas that the large-scale networks (1) share a conserved motif and (2) are embedded within a broad macroscale organization. As a technical point, the present results underscore a need to move from group-based analyses to examination of detailed anatomy within the individual.

Canonical Networks Fractionate into Distinct Networks within the Individual

The most pressing finding reported here is that three major networks (DN, FPN, and dATN) are each subdivided into parallel spatially juxtaposed networks (Figures 1, 3, 4, and 5). Regions

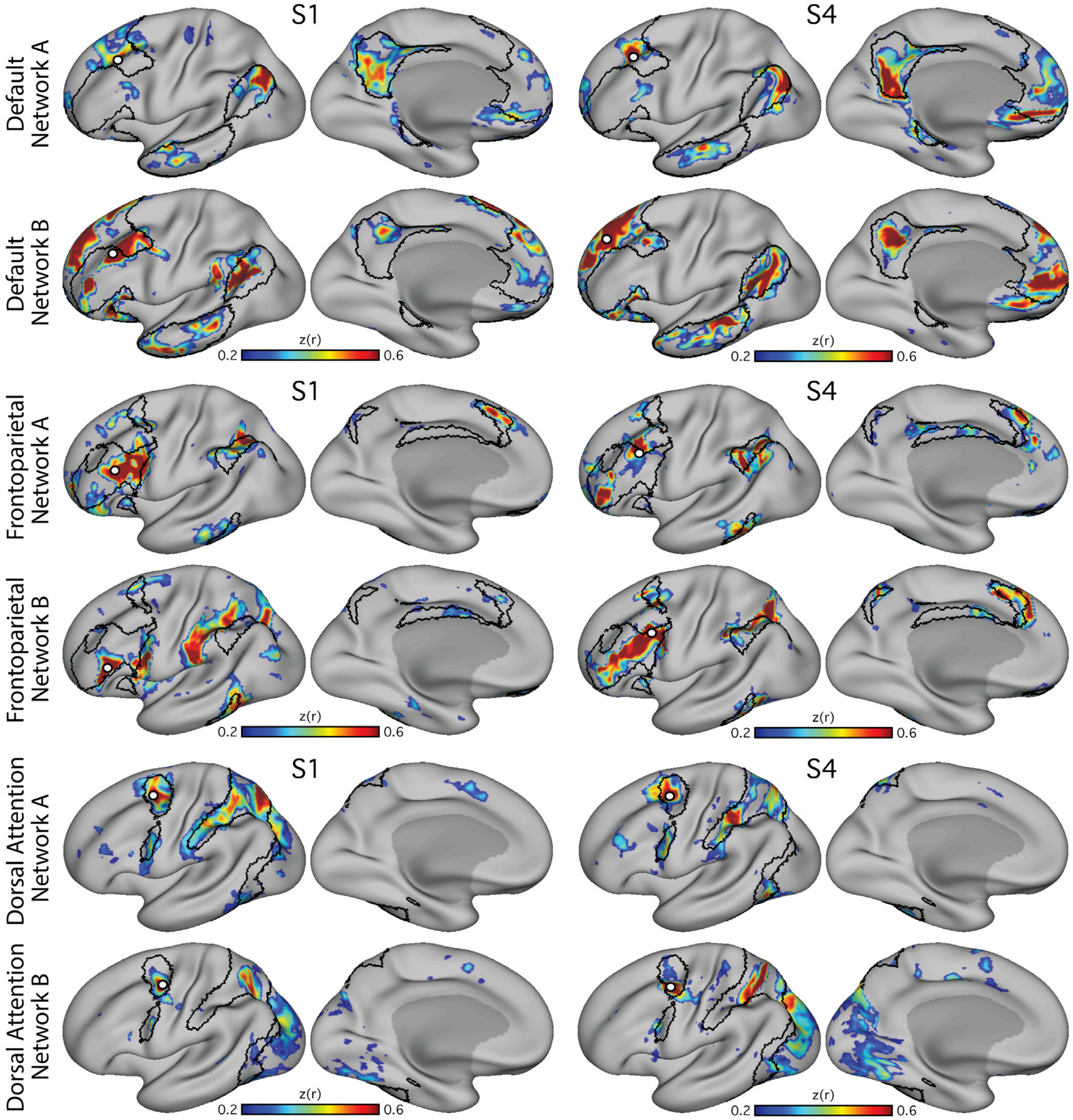

Figure 5. Relationship of Parallel Interdigitated Networks to Canonical Networks from Group-Averaged Data

Each row illustrates how the networks identified in two individuals (S1 and S4) correspond to the well-characterized topography of group-derived networks. The black border represents the outline of the canonical default, frontoparietal control, and dorsal attention networks (top to bottom) calculated using data from 1,000 subjects that were parcellated into seven networks (from Yeo et al., 2011). The correlation maps from each seed (white filled circle) are shown in color. Broadly, the networks can be seen to occupy separate, closely juxtaposed regions that fall within the canonical network borders in most cases. Exceptions can also be found, such as in the inferior frontal cortex in Default Network A and in the parietal lobe in Frontoparietal Control Network B, where the individual's connectivity map strays outside the group network borders.

of the separate networks lay side by side to one another across several cortical zones (Figure 3) and exhibited complex topography, with one network's region sometimes surrounded by another's (Figures 6 and 7). It is thus unsurprising that detailed within-subject analysis is needed to visualize the separate networks. We focused on the DN first to show that the

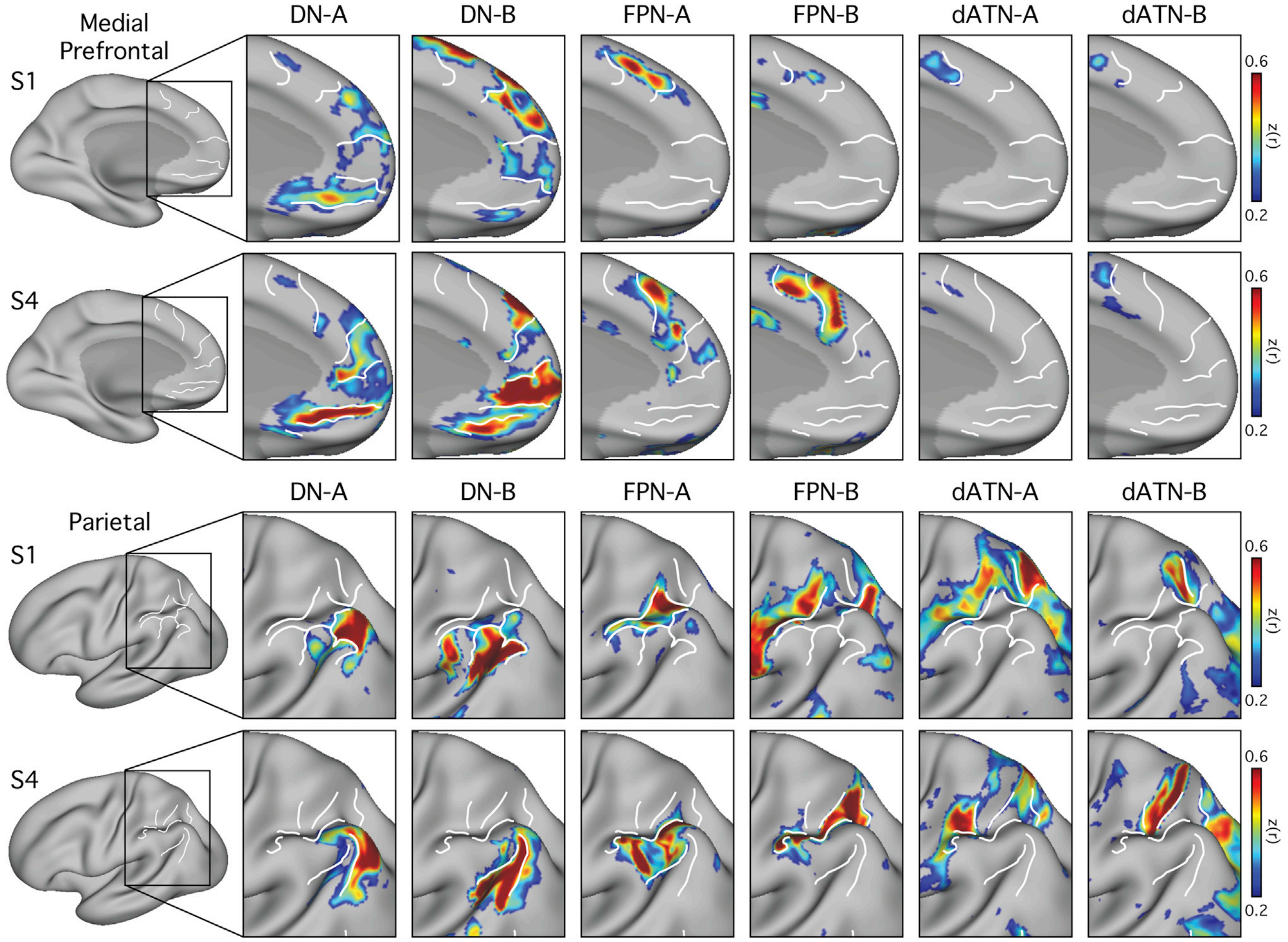

Figure 6. Detailed Anatomy of Six Distinct Networks: Parietal and Medial Prefrontal Cortices

The fine-scale interdigitation of the six identified networks is highlighted in regions where the macroscale organization is evident. White lines serve as landmarks so that the relative position of each network can be appreciated across panels: Networks A and B of the Default Network (DN-A, DN-B), Frontoparietal Control Network (FPN-A, FPN-B), and Dorsal Attention Network (dATN-A, dATN-B). In each row, FC maps from an individual are displayed for either medial frontal cortex (top two rows) or lateral parietal cortex (bottom two rows).

within-subject network fractionation is reproducible across (Figure 1) and within individuals (Figure S3) and is statistically robust in an independent sample (Figure 2). We next characterized fractionations of the FPN and the dATN (Figures 4, 5, 6, and 7).

These findings raise the prospect that the canonical networks studied in group-averaged data consist of distinct functional networks that are blurred together by spatial averaging. The six networks identified here appear to be fractionations of networks identified in group studies (e.g., Yeo et al., 2011; Power et al., 2011; Doucet et al., 2011). This point is important to emphasize: it is not the case that more detailed analysis carved network organization orthogonally to prior schemes, but rather fractionated the existing lower-resolution frameworks (Figure 5). It remains to be determined whether these different networks mediate separable functions across different task contexts. Such a finding could help explain the heterogeneity in cognitive functions and clinical conditions ascribed to the canonical networks (e.g., Buckner et al., 2008; Spreng et al., 2009; Menon, 2011; Laird et al., 2011; Leech and Sharp, 2014; Andrews-Hanna et al., 2014).

Relations to Prior Observations

There is growing consensus that idiosyncratic features exist within individuals that are not captured (or are attenuated) by examining group central tendencies (e.g., Fedorenko et al., 2010, 2012; Mueller et al., 2013; Laumann et al., 2015; Glasser et al., 2016; Huth et al., 2016; Jakobsen et al., 2016; Gordon et al., 2017a, 2017b). The DN was originally hypothesized based on the distributed pattern of regions that increase activity in passive relative to active non-self-referential tasks (Andreasen et al., 1995; Shulman et al., 1997; Mazoyer et al., 2001; for review, see Gusnard and Raichle, 2001). The DN was later estimated using FC by placing a moderately sized seed region in the center of posteromedial cortex (often labeled posterior cingulate cortex or “PCC”) and plotting the correlation pattern (Greicius et al., 2003). One immediate feature of the resulting network that

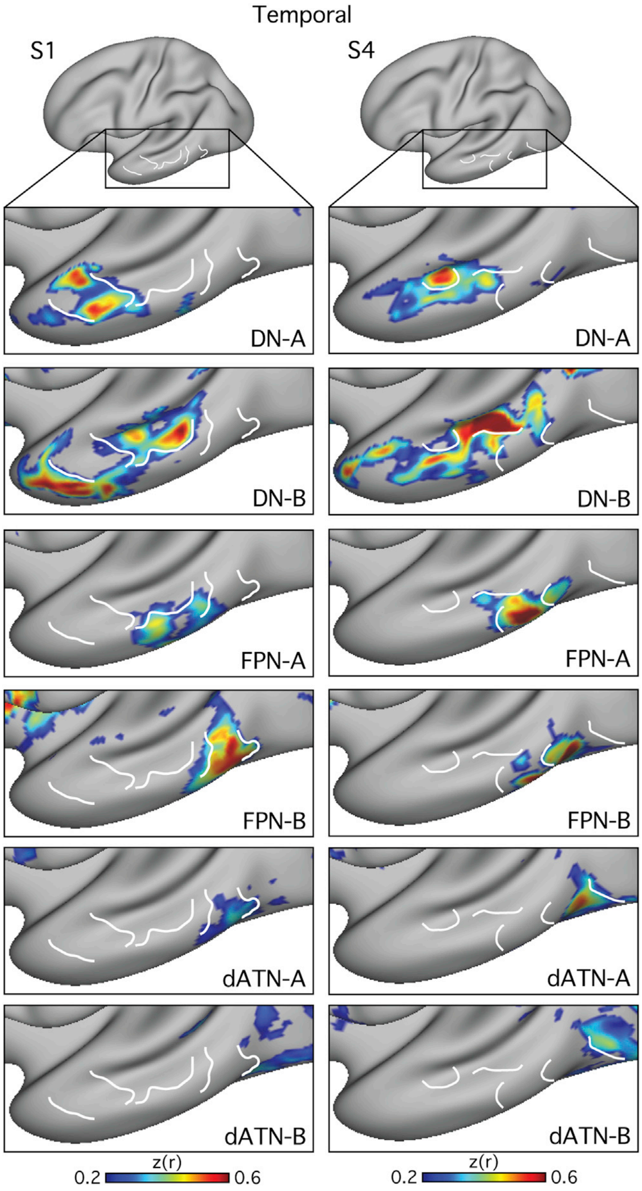

Figure 7. Detailed Anatomy of Six Distinct Networks: Lateral Temporal Cortex

In a similar format to Figure 6, each column displays FC maps from an individual to illustrate the fine-scale interdigitation in the lateral temporal cortex.

raises the possibility of further subdivision is its large size. For example, the canonical group-averaged FC estimate of the DN contains an extensive correlation pattern extending from the dorsal extent of the frontal midline to ventromedial and orbitomedial PFC (e.g., Fox and Raichle, 2007; Buckner et al., 2008).

A number of group-based studies, including our own, have sought to fractionate the DN into subnetworks (e.g., Margulies et al., 2007, 2009; Buckner et al., 2008; Fransson and Marrelec, 2008; Andrews-Hanna et al., 2010, 2014; Leech et al., 2011, 2012). While the present fractionation is not contained within these past efforts, prior observations have noted juxtaposed nodes in different zones of cortex. There is evidence for a dorsal to ventral separation in the posterior midline (Margulies et al., 2009; Leech et al., 2011; see also Vogt et al., 2006) that likely corresponds to the present DN-A and DN-B networks, although the topography is not fully captured by a simple linear axis in all individuals (e.g., see S2 in Figure 1). Prior reports have also noted that a subnetwork of the DN is coupled to the hippocampal formation (e.g., Andrews-Hanna et al., 2010), consistent with anatomical connectivity in the macaque (see Kahn et al., 2008 for review). The present hypothesized DN-A aligns well to the "hippocampal" subnetwork of Andrews-Hanna et al. (2010), while DN-B does not. This separation also informs our understanding of the IPL.

While many analyses of the DN, especially those based on increased response during passive tasks, reveal a large region covering much of IPL, social tasks involving mentalizing (theory-of-mind tasks) activate the more anterior temporoparietal junction (TPJ; Saxe and Kanwisher, 2003) while tasks involving episodic remembering activate a caudal region (e.g., Andrews-Hanna et al., 2014) near what might be a monkey homolog of Opt (Pandya and Seltzer, 1982; for discussion, see Yeo et al., 2011). Group-level FC analyses have noted that the posterior portion of IPL is preferentially coupled to the PHC, while the anterior portion is not (see Yeo et al., 2011's Figure 30). Furthermore, monkey anatomical tracing studies consistently show that PHC projects to a circumscribed portion of area 7A within Opt (e.g., Lavenex et al., 2002, Case M-2-90; Blatt et al., 2003, Cases 1 and 5). These collective findings are consistent with the distinction between DN-A, which is coupled to the PHC and posterior IPL, and DN-B, which involves a more anterior IPL region. What is novel in the present work is that this distinction is now shown to be one spatial component of a much broader separation of two parallel large-scale distributed networks.

We know of no precedent for one feature of our results. The ventral portion of the frontal midline is considered a projection zone of limbic structures, including the amygdala (Ongür and Price, 2000) and hippocampal formation (Rosene and Van Hoesen, 1977). However, in the present study, the most ventral representation of DN-B is inferior to DN-A. This is unexpected because DN-B is differentiated from DN-A by its absence of coupling to the PHC and retrosplenial cortex (Figure S2). This may indicate that a subregion of ventromedial PFC is tied to a large-scale distributed network that is functionally separated from a direct limbic influence. The fine interdigitation shown in Figure 6 reveals why group averaging will likely blur the two networks. In each individual, there are multiple regions for each network that are interposed and whose positions spatially shift between individuals. Anatomical tracing in the monkey will be needed to substantiate whether there exists a ventral midline region that is minimally connected to limbic structures.

Parallel Large-Scale Distributed Networks Are an Organizing Principle of Association Cortex

A notable feature of the identified networks is that each contains components in frontal, parietal, temporal, and frontal midline regions. This repeating pattern or motif has been discussed previously in group data (e.g., Yeo et al., 2011; Power et al., 2011).

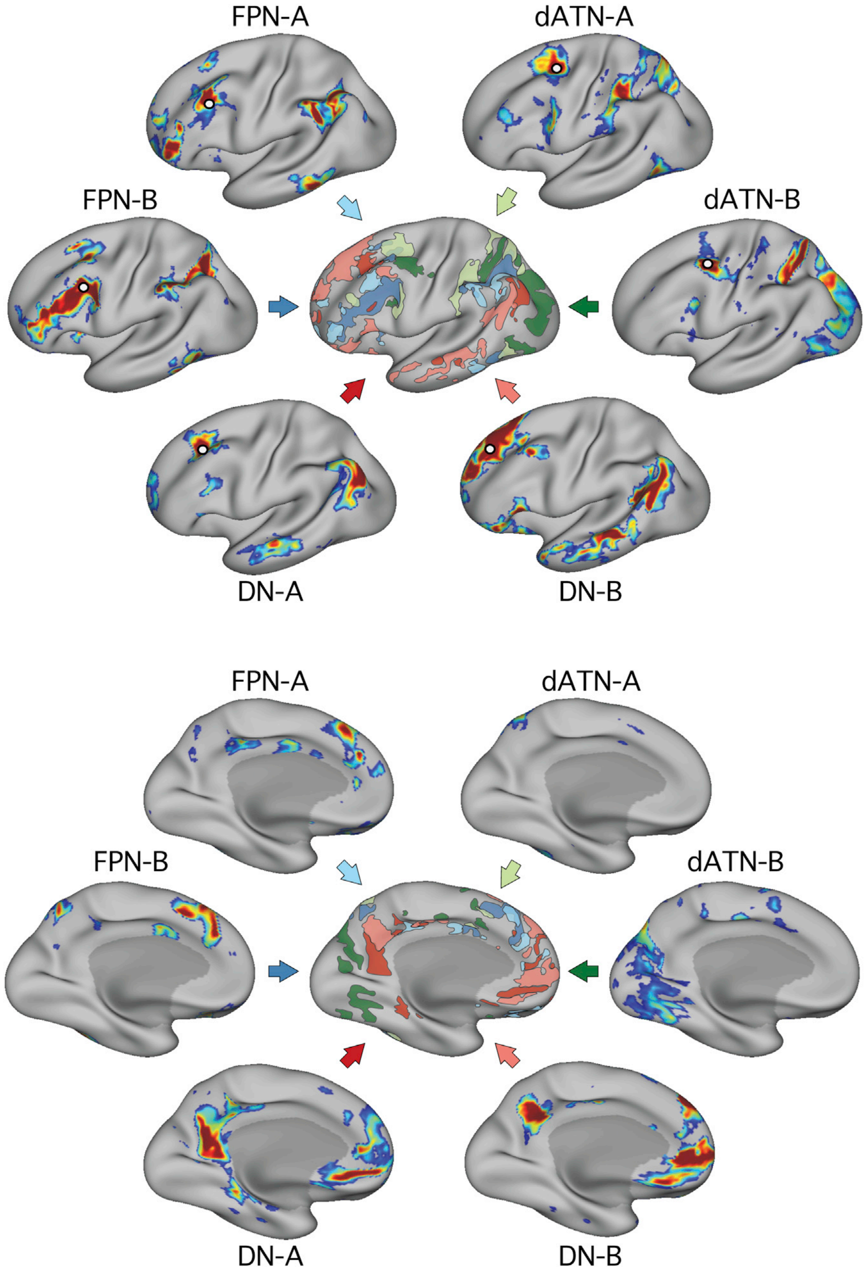

Figure 8. Diagrammatic Representation of Six Parallel Distributed Networks within One Individual

The central figure shows an illustration of the six networks overlaid on the same cortical surface. The top panel shows the lateral view, and the lower panel shows the medial view. The different colors correspond to the canonical network that each network resembles (red, Default Network, DN-A and DN-B; blue, Frontoparietal Network, FPN-A and FPN-B; green, Dorsal Attention Network, dATN-A and dATN-B). The names of the networks are based on prior literature, recognizing that the novel organization identified here may lead to a reconsideration of the functional domains. Data shown are from S4 (see also Figure S6).

What is striking is how well the same distributed pattern accounts for much of the newly fractionated networks.

Goldman-Rakic (1988) suggested that this distributed motif was a general organizing principle of association circuits. Based on results from double-labeling tracer injections by Selemon and Goldman-Rakic (1985, 1988), she posited (1) that prefrontal and parietal areas are embedded within densely interconnected distributed circuits that include midline and temporal areas and (2) that this same motif repeats across nearby zones forming closely adjacent parallel networks. Our results are consistent with her ideas.

One interesting finding is that there can be clear distinctions even between networks with closely juxtaposed regions. As noted, DN-B does not couple to the hippocampal formation, while DN-A does. Similarly, dATN-B shows coupling to retinotopic visual regions along the midline, preferentially to the peripheral field representation, while dATN-A does not (see Figure S8). The presence of such differences may shed insight into the functional role of the parallel networks and their development. One possibility is that there are broad constraints that establish the same motif but, seeded by competing inputs from limbic and sensory systems, activity-dependent processes differentiate the networks during development.

A second important finding is the fine spatial scale that differentiates neighboring networks. The spatial interdigitation of distinct regions was notable. In the lateral temporal lobe, one network's region could be surrounded by another's. This fine-scale interdigitation has implications for interpreting prior network estimations. For example, Mesulam (1981) proposed a cortical network important to spatial attention. Extensive findings illustrate that certain areas near to the intraparietal sulcus form part of a sensory-motor hierarchy, sharing reciprocal projections with extrastriate visual cortex and the frontal eye fields (Maunsell and van Essen, 1983; Ungerleider and Desimone, 1986; Andersen et al., 1990; Boussaoud et al., 1990). However, Mesulam's ideas drew on injections within the IPL area 7a in or near Opt (e.g., Mesulam et al., 1977). This specific parietal association area is near to macaque area LIP but has a projection fingerprint that spares distant extrastriate areas while including cingulate and PHC (Andersen et al., 1990). Given the present results, it is reasonable to suppose that past analyses of parietal association cortex may have lumped together injections in separate parallel, distributed networks.

Evidence for a Macroscale Organization of Association Cortex that Spans Networks

An intriguing finding is revealed when the spatial relations across all the networks are considered together (Figures 8 and S6). While the networks have complex interdigitated relationships, there are also macroscale gradients that share the same general progression in multiple zones of cortex. In parietal association cortex, there is a caudal to rostral progression from DN-A through to dATN-B (Figure 6). In temporal association cortex, there is a rostral to caudal progression through the same networks (Figure 7). The axis is imperfect, and it is sometimes unclear which network should be ordered before the other, but the general ordering that repeats across distinct zones suggests a broad macroscale organizing principle.

Margulies et al. (2016) recently argued that association cortex possesses a macroscale gradient of networks from sensory-motor networks on one end to the DN on the other. In agreement, the possibility was recently raised that association networks evolved from a prototypical distributed sensory-motor network followed by a period of cortical expansion, which freed up zones of association cortex from the constraints of primary sensory input (Buckner and Krienen, 2013; Krienen and Buckner, 2017). The parallel and sequential nature of the presently defined networks adds further support to these ideas.

Limitations and Technical Considerations

An assumption behind our interpretation of the results is that FC across distributed regions is sufficiently constrained by direct and polysynaptic anatomical circuits to provide insight into the organization of distributed networks. Parallels with macaque anatomy reinforce this assumption. However, details of the results may be revised to the degree that factors beyond stable anatomical constraints contribute to the patterns. Exploration of monkey anatomy using multiple tracer injections from adjacent regions in the same animal would be a valuable complement to the present work. The anatomical origin of the fine spatial details are important to resolve because of their implications for clinical endeavors, including presurgical planning and targeted neuromodulation. The present results suggest that targeted neuromodulation of nearby cortical zones could have distinct effects because they are embedded within anatomically separate networks. While this general notion has been appreciated previously (e.g., Fox et al., 2014), what is surprising is the fine spatial scale by which cortical zones participating in distinct networks are interdigitated.

Given that we were able to fractionate established large-scale networks by pushing the practical resolution of fMRI by targeting the individual, it seems likely that our present estimates might also be fractionated further if higher resolution was achieved. Despite efforts to minimize spatial blurring, we were unable to confidently delineate all networks in all individuals. Two subjects, S2 and S3, produced maps that were noticeably blurrier. This difference may be due to several factors, such as differences in head motion, SNR, and misregistration. The reported summary measures do not clearly indicate a cause. The fractionation of the DN into two parallel, distributed networks proved to be the most robust finding observed clearly in all subjects.

The present study is also limited in that we studied only cerebral cortical organization. Ongoing work is characterizing the topography of these networks in subcortical and cerebellar structures.

Conclusions

The present work reveals that there are parallel large-scale distributed networks that are spatially juxtaposed across the cerebral cortex. The spatial scale of these networks is such that they become evident only when analyzed within the individual. Discovery of the presence and description of details of these networks provide a foundation for future study of their functions.

STAR★METHODS

Detailed methods are provided in the online version of this paper and include the following:

- KEY RESOURCES TABLE
- CONTACT FOR REAGENT AND RESOURCE SHARING
- EXPERIMENTAL MODEL AND SUBJECT DETAILS
  - Participants
- METHOD DETAILS
  - MRI Data Acquisition
  - Data Preprocessing
  - Discovery of Networks within the Individual
  - Hypothesis Testing to Dissociate Networks within the Individual
  - Effects of Misalignment between Individuals
  - Additional Explorations Targeting Adjacent Networks within the Individual
  - Confirmation of Interdigitated Network Representations in the Brain Volume
  - Confirmation of Observed Parallel Networks Using Data-Driven Network Parcellation
  - Additional Exploration of Network Organization along Regions of the Medial Temporal Lobe with Low Signal-To-Noise Ratio
  - Test-Retest Reliability of Parallel Interdigitated Networks
- QUANTIFICATION AND STATISTICAL ANALYSIS

SUPPLEMENTAL INFORMATION

Supplemental Information includes eight figures and can be found with this article online at http://dx.doi.org/10.1016/j.neuron.2017.06.038.

AUTHOR CONTRIBUTIONS

R.M.B and R.L.B. designed the study, analyzed the data, interpreted the experiments, and wrote the paper. R.M.B. collected the data.

ACKNOWLEDGMENTS

We thank the Harvard Center for Brain Science neuroimaging core and FAS Division of Research Computing. R. Mair and S. McMains assisted in optimizing acquisition. L. Farfel, M. Marotta, and R.M. Hutchison assisted in data acquisition. R.M.H. and T.M. O'Keefe assisted in data preprocessing. R.M.B. was supported by Wellcome Trust grant 103980/Z/14/Z. This work was supported by Kent and Liz Dauten, NIH grant P50MH106435, and Shared Instrumentation Grant S10OD020039. The multi-band EPI sequence was generously provided by the Center for Magnetic Resonance Research (CMRR) at University of Minnesota.

Received: February 1, 2017
Revised: April 28, 2017
Accepted: June 23, 2017
Published: July 19, 2017

REFERENCES

Amunts, K., Schleicher, A., Bürgel, U., Mohlberg, H., Uylings, H.B., and Zilles, K. (1999). Broca's region revisited: cytoarchitecture and intersubject variability. J. Comp. Neurol. *412*, 319–341.

Amunts, K., Malikovic, A., Mohlberg, H., Schormann, T., and Zilles, K. (2000). Brodmann's areas 17 and 18 brought into stereotaxic space-where and how variable? Neuroimage *11*, 66–84.

Andersen, R.A., Asanuma, C., Essick, G., and Siegel, R.M. (1990). Corticocortical connections of anatomically and physiologically defined subdivisions within the inferior parietal lobule. J. Comp. Neurol. *296*, 65–113.

Andreasen, N.C., O'Leary, D.S., Cizadlo, T., Arndt, S., Rezai, K., Watkins, G.L., Ponto, L.L., and Hichwa, R.D. (1995). Remembering the past: two facets of episodic memory explored with positron emission tomography. Am. J. Psychiatry *152*, 1576–1585.

Andrews-Hanna, J.R., Reidler, J.S., Sepulcre, J., Poulin, R., and Buckner, R.L. (2010). Functional-anatomic fractionation of the brain's default network. Neuron *65*, 550–562.

Andrews-Hanna, J.R., Saxe, R., and Yarkoni, T. (2014). Contributions of episodic retrieval and mentalizing to autobiographical thought: evidence from functional neuroimaging, resting-state connectivity, and fMRI meta-analyses. Neuroimage *91*, 324–335.

Beckmann, C.F., DeLuca, M., Devlin, J.T., and Smith, S.M. (2005). Investigations into resting-state connectivity using independent component analysis. Philos. Trans. R. Soc. Lond. B Biol. Sci. *360*, 1001–1013.

Binder, J.R., Desai, R.H., Graves, W.W., and Conant, L.L. (2009). Where is the semantic system? A critical review and meta-analysis of 120 functional neuroimaging studies. Cereb. Cortex *19*, 2767–2796.

Biswal, B., Yetkin, F.Z., Haughton, V.M., and Hyde, J.S. (1995). Functional connectivity in the motor cortex of resting human brain using echo-planar MRI. Magn. Reson. Med. *34*, 537–541.

Blatt, G.J., Pandya, D.N., and Rosene, D.L. (2003). Parcellation of cortical afferents to three distinct sectors in the parahippocampal gyrus of the rhesus monkey: an anatomical and neurophysiological study. J. Comp. Neurol. *466*, 161–179.

Boussaoud, D., Ungerleider, L.G., and Desimone, R. (1990). Pathways for motion analysis: cortical connections of the medial superior temporal and fundus of the superior temporal visual areas in the macaque. J. Comp. Neurol. *296*, 462–495.

Braga, R.M., and Leech, R. (2015). Echoes of the brain: local-scale representation of whole-brain functional networks within transmodal cortex. Neuroscientist *21*, 540–551.

Buckner, R.L., and Krienen, F.M. (2013). The evolution of distributed association networks in the human brain. Trends Cogn. Sci. *17*, 648–665.

Buckner, R.L., Andrews-Hanna, J.R., and Schacter, D.L. (2008). The brain's default network: anatomy, function, and relevance to disease. Ann. N Y Acad. Sci. *1124*, 1–38.

Buckner, R.L., Krienen, F.M., and Yeo, B.T. (2013). Opportunities and limitations of intrinsic functional connectivity MRI. Nat. Neurosci. *16*, 832–837.

Caspers, S., Geyer, S., Schleicher, A., Mohlberg, H., Amunts, K., and Zilles, K. (2006). The human inferior parietal cortex: cytoarchitectonic parcellation and interindividual variability. Neuroimage *33*, 430–448.

Corbetta, M., and Shulman, G.L. (2002). Control of goal-directed and stimulus-driven attention in the brain. Nat. Rev. Neurosci. *3*, 201–215.

Cowan, W.M., Gottlieb, D.I., Hendrickson, A.E., Price, J.L., and Woolsey, T.A. (1972). The autoradiographic demonstration of axonal connections in the central nervous system. Brain Res. *37*, 21–51.

De Luca, M., Beckmann, C.F., De Stefano, N., Matthews, P.M., and Smith, S.M. (2006). fMRI resting state networks define distinct modes of long-distance interactions in the human brain. Neuroimage *29*, 1359–1367.

Doucet, G., Naveau, M., Petit, L., Delcroix, N., Zago, L., Crivello, F., Jobard, G., Tzourio-Mazoyer, N., Mazoyer, B., Mellet, E., and Joliot, M. (2011). Brain activity at rest: a multiscale hierarchical functional organization. J. Neurophysiol. *105*, 2753–2763.

Evans, A.C., Marrett, S., Neelin, P., Collins, L., Worsley, K., Dai, W., Milot, S., Meyer, E., and Bub, D. (1992). Anatomical mapping of functional activation in stereotactic coordinate space. Neuroimage *1*, 43–53.

Fedorenko, E., Hsieh, P.J., Nieto-Castañón, A., Whitfield-Gabrieli, S., and Kanwisher, N. (2010). New method for fMRI investigations of language: defining ROIs functionally in individual subjects. J. Neurophysiol. *104*, 1177–1194.

Fedorenko, E., Duncan, J., and Kanwisher, N. (2012). Language-selective and domain-general regions lie side by side within Broca's area. Curr. Biol. *22*, 2059–2062.

Felleman, D.J., and Van Essen, D.C. (1991). Distributed hierarchical processing in the primate cerebral cortex. Cereb. Cortex *1*, 1–47.

Fischl, B. (2012). FreeSurfer. Neuroimage *62*, 774–781.

Fischl, B., Sereno, M.I., and Dale, A.M. (1999). Cortical surface-based analysis. II: inflation, flattening, and a surface-based coordinate system. Neuroimage *9*, 195–207.

Fischl, B., Rajendran, N., Busa, E., Augustinack, J., Hinds, O., Yeo, B.T.T., Mohlberg, H., Amunts, K., and Zilles, K. (2008). Cortical folding patterns and predicting cytoarchitecture. Cereb. Cortex *18*, 1973–1980.

Fox, M.D., and Raichle, M.E. (2007). Spontaneous fluctuations in brain activity observed with functional magnetic resonance imaging. Nat. Rev. Neurosci. *8*, 700–711.

Fox, P.T., Perlmutter, J.S., and Raichle, M.E. (1985). A stereotactic method of anatomical localization for positron emission tomography. J. Comput. Assist. Tomogr. *9*, 141–153.

Fox, M.D., Corbetta, M., Snyder, A.Z., Vincent, J.L., and Raichle, M.E. (2006). Spontaneous neuronal activity distinguishes human dorsal and ventral attention systems. Proc. Natl. Acad. Sci. USA *103*, 10046–10051.

Fox, M.D., Buckner, R.L., Liu, H., Chakravarty, M.M., Lozano, A.M., and Pascual-Leone, A. (2014). Resting-state networks link invasive and noninvasive brain stimulation across diverse psychiatric and neurological diseases. Proc. Natl. Acad. Sci. USA *111*, E4367–E4375.

Fransson, P., and Marrelec, G. (2008). The precuneus/posterior cingulate cortex plays a pivotal role in the default mode network: Evidence from a partial correlation network analysis. Neuroimage *42*, 1178–1184.

Friston, K.J., Ashburner, J., Frith, C.D., Poline, J.B., Heather, J.D., and Frackowiak, R.S.J. (1995). Spatial registration and normalization of images. Hum. Brain Mapp. *3*, 165–189.

Geschwind, N. (1965a). Disconnexion syndromes in animals and man. I. Brain *88*, 237–294.

Geschwind, N. (1965b). Disconnexion syndromes in animals and man. II. Brain *88*, 585–644.

Glasser, M.F., Sotiropoulos, S.N., Wilson, J.A., Coalson, T.S., Fischl, B., Andersson, J.L., Xu, J., Jbabdi, S., Webster, M., Polimeni, J.R., et al.; WU-Minn HCP Consortium (2013). The minimal preprocessing pipelines for the Human Connectome Project. Neuroimage *80*, 105–124.

Glasser, M.F., Coalson, T.S., Robinson, E.C., Hacker, C.D., Harwell, J., Yacoub, E., Ugurbil, K., Andersson, J., Beckmann, C.F., Jenkinson, M., et al. (2016). A multi-modal parcellation of human cerebral cortex. Nature *536*, 171–178.

Goldman-Rakic, P.S. (1988). Topography of cognition: parallel distributed networks in primate association cortex. Annu. Rev. Neurosci. *11*, 137–156.

Gordon, E.M., Laumann, T.O., Adeyemo, B., and Petersen, S.E. (2017a). Individual variability of the system-level organization of the human brain. Cereb. Cortex *27*, 386–399.

Gordon, E.M., Laumann, T.O., Adeyemo, B., Gilmore, A.W., Nelson, S.M., Dosenbach, N.U., and Petersen, S.E. (2017b). Individual-specific features of brain systems identified with resting state functional correlations. Neuroimage *146*, 918–939.

Greicius, M.D., Krasnow, B., Reiss, A.L., and Menon, V. (2003). Functional connectivity in the resting brain: a network analysis of the default mode hypothesis. Proc. Natl. Acad. Sci. USA *100*, 253–258.

Greicius, M.D., Srivastava, G., Reiss, A.L., and Menon, V. (2004). Default-mode network activity distinguishes Alzheimer's disease from healthy aging: evidence from functional MRI. Proc. Natl. Acad. Sci. USA *101*, 4637–4642.

Greve, D.N., and Fischl, B. (2009). Accurate and robust brain image alignment using boundary-based registration. Neuroimage *48*, 63–72.

Gusnard, D.A., and Raichle, M.E. (2001). Searching for a baseline: functional imaging and the resting human brain. Nat. Rev. Neurosci. *2*, 685–694.

Henssen, A., Zilles, K., Palomero-Gallagher, N., Schleicher, A., Mohlberg, H., Gerboga, F., Eickhoff, S.B., Bludau, S., and Amunts, K. (2016). Cytoarchitecture and probability maps of the human medial orbitofrontal cortex. Cortex *75*, 87–112.

Hill, J., Inder, T., Neil, J., Dierker, D., Harwell, J., and Van Essen, D. (2010). Similar patterns of cortical expansion during human development and evolution. Proc. Natl. Acad. Sci. USA *107*, 13135–13140.

Holmes, A.J., Hollinshead, M.O., O'Keefe, T.M., Petrov, V.I., Fariello, G.R., Wald, L.L., Fischl, B., Rosen, B.R., Mair, R.W., Roffman, J.L., et al. (2015). Brain Genomics Superstruct Project initial data release with structural, functional, and behavioral measures. Sci. Data *2*, 150031.

Huth, A.G., de Heer, W.A., Griffiths, T.L., Theunissen, F.E., and Gallant, J.L. (2016). Natural speech reveals the semantic maps that tile human cerebral cortex. Nature *532*, 453–458.

Jakobsen, E., Liem, F., Klados, M.A., Bayrak, Ş., Petrides, M., and Margulies, D.S. (2016). Automated individual-level parcellation of Broca's region based on functional connectivity. Neuroimage. Published online September 30, 2016. http://dx.doi.org/10.1016/j.neuroimage.2016.09.069.

Jenkinson, M., Bannister, P., Brady, M., and Smith, S. (2002). Improved optimization for the robust and accurate linear registration and motion correction of brain images. Neuroimage *17*, 825–841.

Jones, E.G., and Powell, T.P. (1970). An anatomical study of converging sensory pathways within the cerebral cortex of the monkey. Brain *93*, 793–820.

Kahn, I., Andrews-Hanna, J.R., Vincent, J.L., Snyder, A.Z., and Buckner, R.L. (2008). Distinct cortical anatomy linked to subregions of the medial temporal lobe revealed by intrinsic functional connectivity. J. Neurophysiol. *100*, 129–139.

Krienen, F.M., and Buckner, R.L. (2017). Human association cortex: expanded, untethered, neotenous, and plastic. In Evolution of Nervous Systems 2e, *Volume 4*, J. Kaas, ed. (Elsevier), pp. 169–183.

Laird, A.R., Fox, P.M., Eickhoff, S.B., Turner, J.A., Ray, K.L., McKay, D.R., Glahn, D.C., Beckmann, C.F., Smith, S.M., and Fox, P.T. (2011). Behavioral interpretations of intrinsic connectivity networks. J. Cogn. Neurosci. *23*, 4022–4037.

Laumann, T.O., Gordon, E.M., Adeyemo, B., Snyder, A.Z., Joo, S.J., Chen, M.-Y., Gilmore, A.W., McDermott, K.B., Nelson, S.M., Dosenbach, N.U.F., et al. (2015). Functional system and areal organization of a highly sampled individual human brain. Neuron *87*, 657–670.

Lavenex, P., Suzuki, W.A., and Amaral, D.G. (2002). Perirhinal and parahippocampal cortices of the macaque monkey: projections to the neocortex. J. Comp. Neurol. *447*, 394–420.

Leech, R., and Sharp, D.J. (2014). The role of the posterior cingulate cortex in cognition and disease. Brain *137*, 12–32.

Leech, R., Kamourieh, S., Beckmann, C.F., and Sharp, D.J. (2011). Fractionating the default mode network: distinct contributions of the ventral and dorsal posterior cingulate cortex to cognitive control. J. Neurosci. *31*, 3217–3224.

Leech, R., Braga, R., and Sharp, D.J. (2012). Echoes of the brain within the posterior cingulate cortex. J. Neurosci. *32*, 215–222.

Marcus, D.S., Harwell, J., Olsen, T., Hodge, M., Glasser, M.F., Prior, F., Jenkinson, M., Laumann, T., Curtiss, S.W., and Van Essen, D.C. (2011). Informatics and data mining tools and strategies for the human connectome project. Front. Neuroinform. *5*, 4.

Margulies, D.S., Kelly, A.M., Uddin, L.Q., Biswal, B.B., Castellanos, F.X., and Milham, M.P. (2007). Mapping the functional connectivity of anterior cingulate cortex. Neuroimage *37*, 579–588.

Margulies, D.S., Vincent, J.L., Kelly, C., Lohmann, G., Uddin, L.Q., Biswal, B.B., Villringer, A., Castellanos, F.X., Milham, M.P., and Petrides, M. (2009). Precuneus shares intrinsic functional architecture in humans and monkeys. Proc. Natl. Acad. Sci. USA *106*, 20069–20074.

Margulies, D.S., Ghosh, S.S., Goulas, A., Falkiewicz, M., Huntenburg, J.M., Langs, G., Bezgin, G., Eickhoff, S.B., Castellanos, F.X., Petrides, M., et al. (2016). Situating the default-mode network along a principal gradient of macroscale cortical organization. Proc. Natl. Acad. Sci. USA *113*, 12574–12579.

Markov, N.T., Vezoli, J., Chameau, P., Falchier, A., Quilodran, R., Huissoud, C., Lamy, C., Misery, P., Giroud, P., Ullman, S., et al. (2014). Anatomy of hierarchy: feedforward and feedback pathways in macaque visual cortex. J. Comp. Neurol. *522*, 225–259.

Maunsell, J.H., and van Essen, D.C. (1983). The connections of the middle temporal visual area (MT) and their relationship to a cortical hierarchy in the macaque monkey. J. Neurosci. *3*, 2563–2586.

Mazoyer, B., Zago, L., Mellet, E., Bricogne, S., Etard, O., Houdé, O., Crivello, F., Joliot, M., Petit, L., and Tzourio-Mazoyer, N. (2001). Cortical networks for working memory and executive functions sustain the conscious resting state in man. Brain Res. Bull. *54*, 287–298.

Mennes, M., Jenkinson, M., Valabregue, R., Buitelaar, J.K., Beckmann, C., and Smith, S. (2014). Optimizing full-brain coverage in human brain MRI through population distributions of brain size. Neuroimage *98*, 513–520.

Menon, V. (2011). Large-scale brain networks and psychopathology: a unifying triple network model. Trends Cogn. Sci. *15*, 483–506.

Mesulam, M.M. (1981). A cortical network for directed attention and unilateral neglect. Ann. Neurol. *10*, 309–325.

Mesulam, M.M. (1990). Large-scale neurocognitive networks and distributed processing for attention, language, and memory. Ann. Neurol. *28*, 597–613.

Mesulam, M.M. (1998). From sensation to cognition. Brain *121*, 1013–1052.

Mesulam, M.M., Van Hoesen, G.W., Pandya, D.N., and Geschwind, N. (1977). Limbic and sensory connections of the inferior parietal lobule (area PG) in the rhesus monkey: a study with a new method for horseradish peroxidase histochemistry. Brain Res. *136*, 393–414.

Michalka, S.W., Kong, L., Rosen, M.L., Shinn-Cunningham, B.G., and Somers, D.C. (2015). Short-term memory for space and time flexibly recruit complementary sensory-biased frontal lobe attention networks. Neuron *87*, 882–892.

Moeller, S., Freiwald, W.A., and Tsao, D.Y. (2008). Patches with links: a unified system for processing faces in the macaque temporal lobe. Science *320*, 1355–1359.

Mueller, S., Wang, D., Fox, M.D., Yeo, B.T.T., Sepulcre, J., Sabuncu, M.R., Shafee, R., Lu, J., and Liu, H. (2013). Individual variability in functional connectivity architecture of the human brain. Neuron *77*, 586–595.

Mueller, S., Wang, D., Fox, M.D., Pan, R., Lu, J., Li, K., Sun, W., Buckner, R.L., and Liu, H. (2015). Reliability correction for functional connectivity: theory and implementation. Hum. Brain Mapp. *36*, 4664–4680.

Murphy, K., Birn, R.M., and Bandettini, P.A. (2013). Resting-state fMRI confounds and cleanup. Neuroimage *80*, 349–359.

Ojemann, J.G., Akbudak, E., Snyder, A.Z., McKinstry, R.C., Raichle, M.E., and Conturo, T.E. (1997). Anatomic localization and quantitative analysis of gradient refocused echo-planar fMRI susceptibility artifacts. Neuroimage *6*, 156–167.

Ongür, D., and Price, J.L. (2000). The organization of networks within the orbital and medial prefrontal cortex of rats, monkeys and humans. Cereb. Cortex *10*, 206–219.

Pandya, D.N., and Seltzer, B. (1982). Intrinsic connections and architectonics of posterior parietal cortex in the rhesus monkey. J. Comp. Neurol. *204*, 196–210.

Patel, G.H., Yang, D., Jamerson, E.C., Snyder, L.H., Corbetta, M., and Ferrera, V.P. (2015). Functional evolution of new and expanded attention networks in humans. Proc. Natl. Acad. Sci. USA *112*, 9454–9459.

Power, J.D., Cohen, A.L., Nelson, S.M., Wig, G.S., Barnes, K.A., Church, J.A., Vogel, A.C., Laumann, T.O., Miezin, F.M., Schlaggar, B.L., and Petersen, S.E. (2011). Functional network organization of the human brain. Neuron *72*, 665–678.

Power, J.D., Schlaggar, B.L., and Petersen, S.E. (2014). Studying brain organization via spontaneous fMRI signal. Neuron *84*, 681–696.

Rademacher, J., Caviness, V.S., Jr., Steinmetz, H., and Galaburda, A.M. (1993). Topographical variation of the human primary cortices: implications for neuroimaging, brain mapping, and neurobiology. Cereb. Cortex *3*, 313–329.

Rajkowska, G., and Goldman-Rakic, P.S. (1995). Cytoarchitectonic definition of prefrontal areas in the normal human cortex: II. Variability in locations of areas 9 and 46 and relationship to the Talairach Coordinate System. Cereb. Cortex *5*, 323–337.

Robinson, E.C., Jbabdi, S., Glasser, M.F., Andersson, J., Burgess, G.C., Harms, M.P., Smith, S.M., Van Essen, D.C., and Jenkinson, M. (2014). MSM: a new flexible framework for Multimodal Surface Matching. Neuroimage *100*, 414–426.

Rosene, D.L., and Van Hoesen, G.W. (1977). Hippocampal efferents reach widespread areas of cerebral cortex and amygdala in the rhesus monkey. Science *198*, 315–317.

Saxe, R., and Kanwisher, N. (2003). People thinking about thinking people. The role of the temporo-parietal junction in "theory of mind". Neuroimage *19*, 1835–1842.

Selemon, L.D., and Goldman-Rakic, P.S. (1985). Longitudinal topography and interdigitation of corticostriatal projections in the rhesus monkey. J. Neurosci. *5*, 776–794.

Selemon, L.D., and Goldman-Rakic, P.S. (1988). Common cortical and subcortical targets of the dorsolateral prefrontal and posterior parietal cortices in the rhesus monkey: evidence for a distributed neural network subserving spatially guided behavior. J. Neurosci. *8*, 4049–4068.

Setsompop, K., Gagoski, B.A., Polimeni, J.R., Witzel, T., Wedeen, V.J., and Wald, L.L. (2012). Blipped-controlled aliasing in parallel imaging for simultaneous multislice echo planar imaging with reduced g-factor penalty. Magn. Reson. Med. *67*, 1210–1224.

Shadlen, M.N., and Newsome, W.T. (2001). Neural basis of a perceptual decision in the parietal cortex (area LIP) of the rhesus monkey. J. Neurophysiol. *86*, 1916–1936.

Shulman, G.L., Fiez, J.A., Corbetta, M., Buckner, R.L., Miezin, F.M., Raichle, M.E., and Petersen, S.E. (1997). Common blood flow changes across visual tasks: II. Decreases in cerebral cortex. J. Cogn. Neurosci *9*, 648–663.

Silbersweig, D.A., Stern, E., Frith, C.D., Cahill, C., Schnorr, L., Grootoonk, S., Spinks, T., Clark, J., Frackowiak, R., and Jones, T. (1993). Detection of thirty-second cognitive activations in single subjects with positron emission tomography: a new low-dose H2(15)O regional cerebral blood flow three-dimensional imaging technique. J. Cereb. Blood Flow Metab. *13*, 617–629.

Smith, S.M., Jenkinson, M., Woolrich, M.W., Beckmann, C.F., Behrens, T.E., Johansen-Berg, H., Bannister, P.R., De Luca, M., Drobnjak, I., Flitney, D.E., et al. (2004). Advances in functional and structural MR image analysis and implementation as FSL. Neuroimage 23 (*Suppl 1*), S208–S219.

Smith, S.M., Miller, K.L., Moeller, S., Xu, J., Auerbach, E.J., Woolrich, M.W., Beckmann, C.F., Jenkinson, M., Andersson, J., Glasser, M.F., et al. (2012). Temporally-independent functional modes of spontaneous brain activity. Proc. Natl. Acad. Sci. USA *109*, 3131–3136.

Smith, S.M., Beckmann, C.F., Andersson, J., Auerbach, E.J., Bijsterbosch, J., Douaud, G., Duff, E., Feinberg, D.A., Griffanti, L., Harms, M.P., et al.; WU-Minn HCP Consortium (2013). Resting-state fMRI in the Human Connectome Project. Neuroimage *80*, 144–168.

Spreng, R.N., Mar, R.A., and Kim, A.S. (2009). The common neural basis of autobiographical memory, prospection, navigation, theory of mind, and the default mode: a quantitative meta-analysis. J. Cogn. Neurosci. *21*, 489–510.

Steinmetz, H., and Seitz, R.J. (1991). Functional anatomy of language processing: neuroimaging and the problem of individual variability. Neuropsychologia *29*, 1149–1161.

Torous, J., Kiang, M.V., Lorme, J., and Onnela, J.P. (2016). New tools for new research in psychiatry: a scalable and customizable platform to empower data driven smartphone research. JMIR Ment Health *3*, e16.

Ungerleider, L.G., and Desimone, R. (1986). Cortical connections of visual area MT in the macaque. J. Comp. Neurol. *248*, 190–222.

van der Kouwe, A.J., Benner, T., Fischl, B., Schmitt, F., Salat, D.H., Harder, M., Sorensen, A.G., and Dale, A.M. (2005). On-line automatic slice positioning for brain MR imaging. Neuroimage *27*, 222–230.

van der Kouwe, A.J., Benner, T., Salat, D.H., and Fischl, B. (2008). Brain morphometry with multiecho MPRAGE. Neuroimage *40*, 559–569.

Van Dijk, K.R., Hedden, T., Venkataraman, A., Evans, K.C., Lazar, S.W., and Buckner, R.L. (2010). Intrinsic functional connectivity as a tool for human connectomics: theory, properties, and optimization. J. Neurophysiol. *103*, 297–321.

Van Essen, D.C. (2005). A Population-Average, Landmark- and Surface-based (PALS) atlas of human cerebral cortex. Neuroimage *28*, 635–662.

Van Essen, D.C., Anderson, C.H., and Felleman, D.J. (1992). Information processing in the primate visual system: an integrated systems perspective. Science *255*, 419–423.

Vincent, J.L., Snyder, A.Z., Fox, M.D., Shannon, B.J., Andrews, J.R., Raichle, M.E., and Buckner, R.L. (2006). Coherent spontaneous activity identifies a hippocampal-parietal memory network. J. Neurophysiol. *96*, 3517–3531.

Vincent, J.L., Patel, G.H., Fox, M.D., Snyder, A.Z., Baker, J.T., Van Essen, D.C., Zempel, J.M., Snyder, L.H., Corbetta, M., and Raichle, M.E. (2007). Intrinsic functional architecture in the anaesthetized monkey brain. Nature *447*, 83–86.

Vincent, J.L., Kahn, I., Snyder, A.Z., Raichle, M.E., and Buckner, R.L. (2008). Evidence for a frontoparietal control system revealed by intrinsic functional connectivity. J. Neurophysiol. *100*, 3328–3342.

Vogt, B.A., Vogt, L., and Laureys, S. (2006). Cytology and functionally correlated circuits of human posterior cingulate areas. Neuroimage *29*, 452–466.

Voyvodic, J.T., Glover, G.H., Greve, D., and Gadde, S.; FBIRN (2011). Automated real-time behavioral and physiological data acquisition and display integrated with stimulus presentation for FMRI. Front. Neuroinform. *5*, 27.

Weiskopf, N., Hutton, C., Josephs, O., and Deichmann, R. (2006). Optimal EPI parameters for reduction of susceptibility-induced BOLD sensitivity losses: a whole-brain analysis at 3 T and 1.5 T. Neuroimage *33*, 493–504.

Yeo, B.T.T., Krienen, F.M., Sepulcre, J., Sabuncu, M.R., Lashkari, D., Hollinshead, M., Roffman, J.L., Smoller, J.W., Zöllei, L., Polimeni, J.R., et al. (2011). The organization of the human cerebral cortex estimated by intrinsic functional connectivity. J. Neurophysiol. *106*, 1125–1165.

Yeo, B.T.T., Krienen, F.M., Chee, M.W., and Buckner, R.L. (2014). Estimates of segregation and overlap of functional connectivity networks in the human cerebral cortex. Neuroimage *88*, 212–227.

Zou, Q.H., Zhu, C.Z., Yang, Y., Zuo, X.N., Long, X.Y., Cao, Q.J., Wang, Y.F., and Zang, Y.F. (2008). An improved approach to detection of amplitude of low-frequency fluctuation (ALFF) for resting-state fMRI: fractional ALFF. J. Neurosci. Methods *172*, 137–141.

STAR★METHODS

KEY RESOURCES TABLE

REAGENT or RESOURCE	SOURCE	IDENTIFIER
Software and Algorithms		
FreeSurfer	Fischl, 2012	http://surfer.nmr.mgh.harvard.edu
MATLAB	MathWorks	www.mathworks.com
FSL	Smith et al., 2004	https://fsl.fmrib.ox.ac.uk/fsl/fslwiki/
Connectome Workbench	Marcus et al., 2011	www.humanconnectome.org

CONTACT FOR REAGENT AND RESOURCE SHARING

Further information and requests for resources should be directed to and will be fulfilled by the Lead Contact, Rodrigo M. Braga (rodrigo.braga@imperial.ac.uk or rbraga@fas.harvard.edu).

EXPERIMENTAL MODEL AND SUBJECT DETAILS

Participants

Four healthy female right-handed young adults (ages 21 to 26) were recruited from the greater Boston community for a study that involved 24 separate MRI scanning sessions as well as extensive behavioral monitoring over a period of approximately 16 weeks. None of the participants were local (Harvard) students or institutional employees, and all were paid for participation with milestone payments at the end of the trial period (two weeks), after 12 MRI sessions, and after all 24 MRI sessions. Participants were screened to exclude a history of neurological or psychiatric illness, or ongoing use of psychoactive medications. Seven participants were enrolled and three chose not to continue within the first two weeks (which were described to all participants as a trial period). Four participants were enrolled for the full 24-session extended study and all of these individuals completed all intended sessions. The present paper concerns only the fMRI data, but enrolled individuals also participated in extensive behavioral testing and daily monitoring of behavior via smart phones (Beiwe; Torous et al., 2016), sleep and activity monitoring via an Actigraph 2 wrist wearable (Philips Respironics, Murrysville, PA, USA), as well as hearing tests at several times during the study (Model 2500 Microprocessor Audiometer, AMBCO, Tustin, CA, USA). One subject required vision correction using MRI compatible glasses. Participants provided written informed consent in accordance with the guidelines set by the Institutional Review Board of Harvard University.

METHOD DETAILS

MRI Data Acquisition

Data were collected on a 3T Siemens Prisma-fit MRI scanner (Siemens Healthcare, Erlangen, Germany) using the vendor's 64-channel phased-array head-neck coil. Heads were immobilized with Siemens small foam head coil wedges. Each of the 24 MRI sessions included one 7 m 2 s run of resting state data (passive fixation) to estimate intrinsic functional connectivity (Biswal et al., 1995) as well as a number of other acquisitions (structural, ASL, and task-based functional runs). The resting state run was collected in the same fixed order during every session near to the beginning of the session to optimize compliance. Participants were instructed to remain still, stay awake and to fixate a centrally presented crosshair presented in black on a light gray background. The gray background color was used instead of white to reduce glare, eye fatigue and discomfort. The position of the screen was adjusted at the beginning of each session to ensure a comfortable, central viewing position to minimize muscle tension and head motion. Before each session participants were encouraged to spend a few minutes finding a comfortable lying position that they could maintain for the entire session. The scanner room lights were kept on to deter participants from becoming drowsy.

Eye closures and movements were monitored using the Eyelink 1000 Core Plus with Long-Range Mount. A video of the eye tracker output was recorded in order to quantify compliance and arousal. Additional in-scanner physiological monitoring (Biopac Systems Inc, Goleta, CA, USA) included a respirator belt around the chest to monitor breathing (Biopac, TSD221-MRI), electrodermal electrodes (Biopac, EL508) attached to participants' right sole to measure galvanic skin response, and a band pulse-oximeter (Biopac, OXY-MRI-SENSOR) attached to participants' right middle toe to measure oxygen saturation and pulse rate. See Voyvodic et al. (2011) for details. The oximeter and electrodes were place on participants' feet to keep their hands free to make responses using button boxes during the in-scanner tasks. Immediately before each run, participants were asked to remain still for the entirety of

the upcoming run. Following each run, participants were given feedback about noted movements and encouraged to stay still, in order to establish an expectation that their compliance and movement were being carefully watched.

Functional imaging data were acquired using a multi-band gradient-echo echo-planar pulse sequence (Setsompop et al., 2012) with acquisition parameters: TR 1000 ms; TE 32.6 ms; flip angle 64°; 2.4 mm isotropic voxels; FOV 211 mm x 211 mm x 156 mm; 65 slices fully covering the cerebral cortex and cerebellum. Slice acquisition used interleaved simultaneous multi-slice 5x acceleration. The sequence was a custom sequence generously provided by the Center for Magnetic Resonance Research (CMRR) at University of Minnesota. Whole brain coverage and minimization of signal dropout due to magnetic susceptibility were achieved by aligning slices to a plane 25 degrees from the anterior commissure-posterior commissure plane toward the coronal plane (Weiskopf et al., 2006; Mennes et al., 2014). This was implemented using an automated alignment procedure to ensure consistency across sessions (van der Kouwe et al., 2005) and, in pilot acquisitions, was found to increase signal-to-noise in ventromedial prefrontal cortex (PFC). A rapid T1-weighted structural image was also acquired in each session using a multi-echo MPRAGE three-dimensional sequence (van der Kouwe et al., 2008) with acquisition parameters: TR 2200 ms; TE 1.57, 3.39, 5.21, 7.03 ms; flip angle 7°; 1.2mm isotropic voxels; 144 slices; FOV 230 mm x 230 mm x 173 mm, in-plane GRAPPA acceleration 4 (see Holmes et al., 2015 for empirical results and discussion of comparability of this brief sequence to traditional longer acquisitions).

Data Preprocessing

Resting-state data were processed using methods similar to those previously described (Van Dijk et al., 2010; Yeo et al., 2011): (1) 12 initial volumes from each run were discarded to allow for T1-equillibration, (2) head motion was corrected using rigid body translation and rotation (FSL, https://fsl.fmrib.ox.ac.uk/fsl/fslwiki/; Jenkinson et al., 2002; Smith et al., 2004), (3) data were temporally low-pass filtered at a threshold of 0.08 Hz, and (4) nuisance variables (6 motion parameters, mean whole-brain signal, mean ventricular signal, mean deep cerebral white matter signal) and their temporal derivatives were regressed. Structural data were processed using the FreeSurfer version 4.5.0 software package (http://surfer.nmr.mgh.harvard.edu; Fischl, 2012). For each anatomical image (one per session), a surface mesh representation of the cortex was reconstructed and registered to a common spherical coordinate system by aligning the major sulcal patterns to the FreeSurfer average template (Fischl et al., 1999). The preprocessed functional images from each session were aligned to the cortical surface mesh reconstructed from that session's anatomical image using boundary-based registration (Greve and Fischl, 2009). Functional data were then propagated to the common spherical coordinate system via sampling (trilinear interpolation) from the middle of the cortical ribbon in a single interpolation step.

Functional data were sampled to the fsaverage6 surface mesh (Fischl et al., 1999) containing 40,962 vertices per hemisphere, and a 2mm full-width-at-half-maximum (FWHM) smoothing kernel was applied to the data in the surface space. A mesh resolution of 40,962 vertices was chosen to reduce blurring during the trilinear interpolation step and hence maximize the potential for observing network distinctions, while keeping the computational burden manageable. A bespoke cortical surface template containing 40,962 vertices per hemisphere was produced using the Connectome Workbench's command suite (Glasser et al., 2013). This was done so that the functional connectivity analyses could be performed and visualized interactively within the Workbench's flexible surface-based visualization software, wb_view (Marcus et al., 2011). The bespoke template was created by combining the left and right pial surfaces from the fsaverage6 freesurfer template into the CIFTI format using the Workbench commands. The pial surfaces were then selectively inflated (smoothing cycles: 3, smoothing strength 0.7, smoothing-iterations 13, inflation factor 1.02) using Workbench to allow visualization of the major cortical folds while maintaining the majority of the cortical surface visible.

Voxel-based tSNR maps were computed by taking the motion-corrected time series from each functional run and dividing the mean signal at each voxel by its standard deviation over time. The tSNR maps were then averaged across functional runs within the Discovery (n = 12), Replication (n = 12) and Full datasets (n = 24), and the resulting mean tSNR maps were projected to the cortical surface for visualization using FreeSurfer (Figure S1). An additional metric of data quality, fractional Amplitude of Low Frequency Fluctuations (fALFF), was also computed (Figure S1). fALFF maps were produced by normalizing the total power in the low (0.01 – 0.08 Hz) frequency range by the total power across all frequencies (Zou et al., 2008).

Discovery of Networks within the Individual

For each participant, half of her data were used in a discovery manner to identify networks that would be later tested in the remaining independently collected data sessions. The odd-numbered sessions (i.e., 1st, 3rd, 5th, etc) formed the discovery dataset (n = 12) while the even-numbered sessions (i.e., 2nd, 4th, 6th, etc) were set aside as the hypothesis-testing dataset (next section). The discovery datasets in each of the four participants were analyzed blind to the hypothesis-testing datasets.

For the discovery analysis, Pearson's product moment correlations between the fMRI time series at each cortical surface vertex were computed. This resulted in an 81,924 × 81,924 element cross-correlation matrix (40,962 vertices per hemisphere) for each of the 12 fMRI runs from the discovery dataset. The matrices were then r-to-z transformed and averaged to yield a mean matrix with high stability. These discovery matrices were used to explore network organization. The mean cross correlation matrices were assigned to the bespoke cortical template so that individual seed vertices could be selected and their functional connectivity maps interactively viewed using wb_view (Marcus et al., 2011). Individual vertices were selected from the general vicinity of expected locations of target networks in PFC (as estimated from independent group-averaged data; n = 1000 from Yeo et al., 2011) and expectations from the literature (e.g., Power et al., 2011; Yeo et al., 2011).

First, a seed vertex was selected from lateral PFC near regions which form part of the canonical default network, and the resulting maps visualized. If the functional connectivity map revealed a network of distributed regions showing a robust correlation with the seed region (z(r) $\approx$0.6), the seed vertex number was recorded for further analysis. If the map revealed no strong correlations, or a diffuse network of correlations that were observably lower (z(r) $\approx$0.4), this was taken as evidence that the seed region was sampling an area of signal dropout or containing a mixture of signals, respectively, and a different seed region was selected. The colorbar scale was set between 0.2 and 0.6, using the JET256 palette in wb_view, so that the correlation structure could be adequately represented and these subtle differences observed. After a robust network was detected and recorded, a different seed vertex was selected that satisfied 4 criteria: the second seed region i) was within the lateral PFC, ii) was in the vicinity of the previously identified network's seed, iii) was in a region of the cortical surface that showed low correlation (z(r)<0.3) with the previous network's seed region, and iv) also showed robust correlation with a distributed set of regions. Thus the analysis converged on robust networks that had closely neighboring representations within the lateral PFC.

The goal of this discovery procedure was not to confirm expectations from the group data, but to allow the individual participant maps to be extensively interrogated, moving outward from properties expected from the group maps. Given prior analyses of within-subject data (e.g., Figure 4 from De Luca et al., 2006; Figure 3 from Vincent et al., 2006; Figure 9 from Van Dijk et al., 2010), it was unsurprising that many maps constructed within the individual participants resembled canonical networks discovered in group-analyzed data. The targets of our exploration were network features that are not fully captured by group analyses (e.g., Laumann et al., 2015; Gordon et al., 2017a, 2017b; see also Fedorenko et al., 2012).

As the results will demonstrate, seed regions placed in nearby regions of lateral PFC revealed two important features that led to the regions selected for the hypothesis-testing phase of analysis. The first feature was that, like typical group-based analyses, the resulting distributed networks contained inferior parietal, temporal, medial prefrontal, and posteromedial cortical components near to what has been described as the 'default network' (DN). Second, nearby seed regions yielded distributed networks that were closely neighboring but separate throughout much of the distributed organization of the network. That is, two distinct networks were closely interdigitated throughout the topography of the canonical DN suggesting the hypothesis that the DN may be comprised of multiple neighboring networks. We refer to these hypothesized networks as Network A and Network B (Figure 1).

Hypothesis Testing to Dissociate Networks within the Individual

The discovery phase of data analysis led to the hypothesis that two distinct interdigitated networks exist that each are within or near to the canonical DN (Figure 1). The two networks were present in each individual participant. The goal of the hypothesis-testing phase was to use independent data in each participant to support or refute the possibility of two dissociable networks. To conduct this analysis, two distinct neighboring lateral PFC seed regions were selected within each participant as well as pairs of regions throughout the cortex based only on the discovery datasets that maximized the separation of the distributed networks. A priori regions (single vertices) were selected in each of the main regional zones of the cortex (temporal, inferior parietal, posteromedial, and medial prefrontal). These a priori regions were targeted to locations where contiguous vertices could be observed in Network A and Network B (Figure 1). A seed region was also selected in parahippocampal cortex (PHC) to quantify the representation of Network A in this region, given a prior literature linking the hippocampal formation to the DN (Greicius et al., 2004; Vincent et al., 2006; Kahn et al., 2008). These regions were then statistically tested in the independent data to formally dissociate the two networks.

The critical test was whether there would be significant interactions between the two lateral PFC seed regions and Network A and Network B regions in each of the zones of cortex in the independent hypothesis-testing datasets. The presence of significant interactions would be evidence for regional dissociations. The presence of interactions across all distributed zones of the cortex would be strong evidence that there was a complete network dissociation across the cortex, in essence establishing dissociable but adjacent networks. For each of the 12 hypothesis-testing data sessions within each participant, values representing the r-to-z transformed Pearson's product moment correlation for each of the two lateral PFC seed regions were computed in relation to each of the distributed test regions. Statistical tests were performed as a two-way ANOVA. In each ANOVA, the independent (classification) variables were the PFC seed location (A and B) and the a priori test regions (A and B) within one of the separate zones of cortex. The dependent variable was the r-to-z transformed Pearson's product moment correlation between each seed and test region. Each cell contained a correlation measure from each of the 12 sessions in the hypothesis-testing dataset, producing a balanced 2 X 2 factorial design with 12 elements in each cell. This 2-factor analysis was repeated for each of the separate cortical zones (temporal, inferior parietal, posteromedial, and medial prefrontal). Statistical significance level was set at $p < 0.01$. Critically, the multiple tests across the network and across subjects were not independent in the sense that they were targeting convergent evidence for network dissociation. Thus multiple, repeated significant results in the ANOVA across regions and participants would provide a high level of certainty for dissociation. Sporadic significance that occurred in 1 in 20 or 1 in 100 tests that showed no specific pattern might be indicative of false positives. As the results will reveal, the data patterns and hypothesis-directed statistical tests were quite clear in the weight of their evidence.

Effects of Misalignment between Individuals

The details of network organization that are revealed by our analyses within individuals suggest a level of spatial and anatomical specificity that would likely be lost or underappreciated when central tendencies across participants are extracted in group-averaged data or when details of anatomy in one participant are assumed to apply to another. That intuition can be appreciated visually by

examining Figures 1, 3, 4, 5, 6, and 7, and also in results from other papers (e.g., Figure 7 from Laumann et al., 2015). To formally explore this issue, we asked to what degree network structure present using individually-tailored regions is lost when one person's anatomy is assumed to apply to another person.

For these analyses, in each of the four participants we extracted the hypothesized cortical regions that were components of DN-A and DN-B from the discovery datasets (lateral prefrontal, temporal, inferior parietal, posteromedial, and medial prefrontal, but not the parahippocampal region; Figure 1), yielding 10 individually-tailored regions per subject. These regions were then used to construct a 10 × 10 matrix of correlations in the hypothesis-testing dataset for each participant. To address the question of whether misalignment between individuals affects the results, we recalculated the matrices in each participant using the regions defined in the other three participants (Figure S5). The goal of this analysis is to provide some level of visualization of what is maintained and what is lost when misalignment is present across individuals. Our focus here is on the specific dissociation between DN-A and DN-B as an example of an important feature of functional-anatomical organization (see Gordon et al., 2017a for a conceptually similar analysis performed for algorithmically defined individually-tailored patches). As can be seen in Figure S5, the clustering of individually tailored regions into the two distinct networks breaks down when seed region locations from a different subject are used. This result illustrates that the spatial variation between individuals is enough to obscure the clear and reproducible network dissociation that is uncovered within the individuals.

Additional Explorations Targeting Adjacent Networks within the Individual

The main analyses explored and tested for dissociation of two distinct neighboring networks that fractionate the canonical DN. This was an unexpected result and encouraged further exploration to determine whether there were further network fractionations. For these additional, post hoc explorations the full dataset for each participant was used to dissociate networks. Results are reported for network properties that were evident in at least two separate participants.

Confirmation of Interdigitated Network Representations in the Brain Volume

Surface projection provides a convenient means to visualize cortical organization and the juxtaposition of networks on the continuous surface. However, projection to the surface involves a spatially-nonlinear transformation that can project nearby voxels in the volume to distant positions on the surface (such as occurs when folds near the crowns of separate gyri abut one another). It is thus important to check that the observed interdigitation between the dissociated networks is present in both surface and volume representations and is not the result of the cortical sampling procedure used. Moreover, volume-based analysis will be required for many applied endeavors including presurgical planning and localization for neuromodulation (e.g., transcranial magnetic and direct-current stimulation).

To explore these issues, we reproduced the findings of two dissociated networks linked to the default network in the volume. The functional data from each subject were preprocessed as described for the surface-based pipeline, with the exceptions that smoothing was performed in the volume at 2mm FWHM prior to regression of nuisance variables and bandpass filtering, and that data were not projected to the surface. The first 8 resting state runs from each subject were concatenated in time and AFNI's InstaCorr (R. Cox and Z. Saad, 2010, International Conference on Resting-State Functional Brain Connectivity, conference; https://afni.nimh.nih.gov/afni) software was used to interactively select seed voxels and view the resulting FC maps. Two seed voxels were selected in left lateral PFC that maximized the separation between the two FC map representations in posteromedial cortex, namely a ventral representation in retrosplenial cortex for DN-A, and the dorsal posterior cingulate cortex representation for DN-B. When the two candidate seed regions were identified, the seed voxel locations were recorded and FC maps were produced in all 24 runs of the data for each subject.

Pearson's product moment correlations were calculated between the fMRI time series at each voxel and the seed voxel. The maps were then r-to-z transformed and averaged to produce a single FC map for each of the two seed regions. The question was whether the two parallel networks would retain the critical features observed in the cortical surface when viewed in the volume (Figure S4). More broadly, while the surface-based visualization proved most effective for discovering organizational details, volume-based analysis is important to fully understand the underlying organizational features, including the possibilities that complex geometry of the sulcal patterns and also non-neuronal structures such as vessels may contribute to estimated features of cortical organization.

Confirmation of Observed Parallel Networks Using Data-Driven Network Parcellation

The network dissociations reported were discovered and replicated using seed-based methods within-subjects that generalized across subjects. If the network dissociations are robust, they should also be observable using multiple approaches. That is, the path we initially employed to uncover them should not be the only way to reveal their presence and their specific spatial organization. To investigate whether the fractionation of the default network generalized across approaches, we explored network organization within the individual participants using data-driven clustering techniques.

We concatenated the surface-projected timeseries data from the Discovery dataset (n = 12), and used MATLAB's *kmeans* function (version R2015a; MathWorks, Natick, MA) to parcellate the vertex timeseries into clusters. As discussed extensively in our earlier analysis of group-based network estimates (Yeo et al., 2011), clustering approaches yield multiple solutions to data parcellations at many levels of clustering. Here we explored a relatively low dimensional fractionation (k = 12) that was able to yield the key dissociated networks including many of their distributed subcomponents.

Additional Exploration of Network Organization along Regions of the Medial Temporal Lobe with Low Signal-To-Noise Ratio

As the results unfolded, the DN-A was found to possess a clear representation in the PHC, while no such representation was detected for DN-B. As portions of the medial temporal lobe are susceptible to signal drop-out in fMRI (Ojemann et al., 1997), additional analyses were conducted to try to find evidence for a representation of DN-B in this region. Additional analyses were focused on the subject that showed the most robust network dissociations (S4). First, the FC maps for DN-A and DN-B from the PFC seeds were disattenuated by using the reliability of the functional connectivity maps across runs as an estimate of the signal dropout (Mueller et al., 2015; Figure S2A). This revealed two representations belonging to DN-B in the inferior temporal lobe along zones of susceptibility artifact, but in regions located well outside the PHC. Second, seed vertices were selected in a posterior to anterior progression along the medial temporal lobe, and the resulting FC maps were observed (Figure S2B). This analysis also did not reveal evidence for a representation of DN-B in the vicinity of the representation of DN-A. We also further explored whether other networks might be detected along the inferior and anterior temporal lobe, once signal dropout was partially accounted for. We describe these additional observations as potential avenues for further more detailed investigations as well as a reminder that, even with our numerous steps to increase signal-to-noise through signal averaging and our use of small voxels and acceleration during acquisition, signal loss due to susceptibility artifacts is still a problem in certain zones of cortex.

Test-Retest Reliability of Parallel Interdigitated Networks

The FC maps for DN-A and DN-B were produced from the Discovery and Replication datasets (Figure S3). Additionally, the mean connectivity matrix from the Discovery (n = 12) and Replication (n = 12) datasets were correlated to show the consistency of the connectivity patterns within each subject (S1: $r = 0.88$; S2: $r = 0.95$; S3: $r = 0.87$; S4: $r = 0.94$, all $p < 0.001$).

QUANTIFICATION AND STATISTICAL ANALYSIS

This study includes n = 4 participants, each of which were scanned over 24 fMRI sessions. Each participant's imaging data were divided into discovery (n = 12; odd-numbered runs) and replication (n = 12; even-numbered runs) samples to allow for data exploration and statistical testing using independent data points. Functional connectivity between brain regions was calculated in MATLAB (version 7.4; http://www.mathworks.com; MathWorks, Natick, MA) using Pearson's product moment correlations which were r to z transformed prior to averaging or comparison. Statistical tests were performed as a two-way ANOVA using MATLAB's *anova2* function (version R2015a). Statistical significance was set to $p < 0.01$. Network parcellation was performed using MATLAB's *kmeans* function (version R2015a).

Neuron, Volume *95*

Supplemental Information

Parallel Interdigitated Distributed Networks within the Individual Estimated by Intrinsic Functional Connectivity

Rodrigo M. Braga and Randy L. Buckner

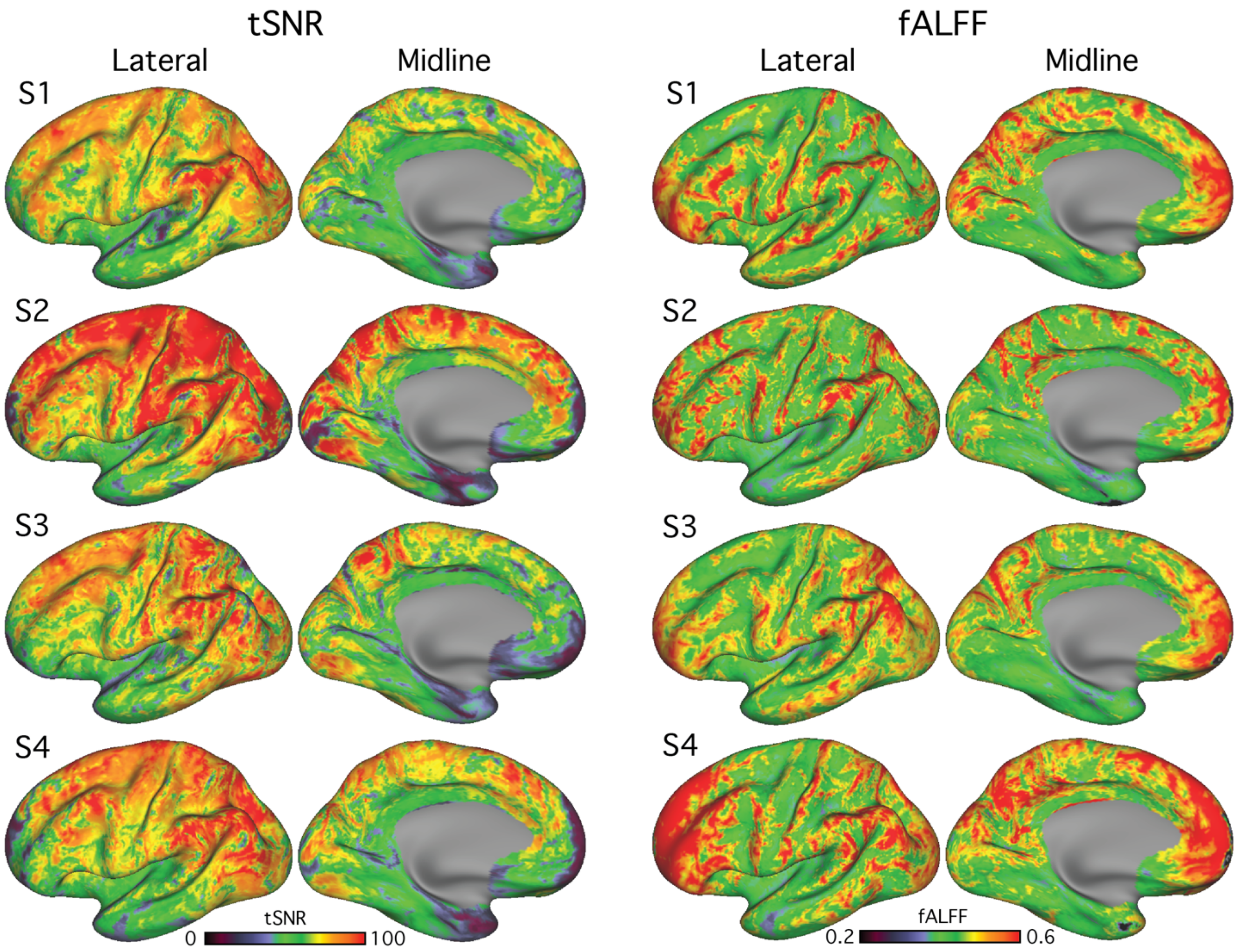

Figure S1: Estimated Temporal Signal-to-Noise Ratio (tSNR) and Fractional Amplitude of Low Frequency Fluctuations (fALFF) of the Functional MRI Data. Related to Figures 1 and 3-8 in the main text. Voxel-based tSNR and fALFF maps created from the full dataset (n = 24) for each subject (S1 – S4) are projected to the cortical surface. The maps demonstrate the spatial distribution of variance in data quality and intensity of regional spontaneous fluctuations across the cortex. While both tSNR and fALFF are decreased in regions of ventromedial prefrontal cortex and the anterior temporal lobe, there is nonetheless considerable coverage.

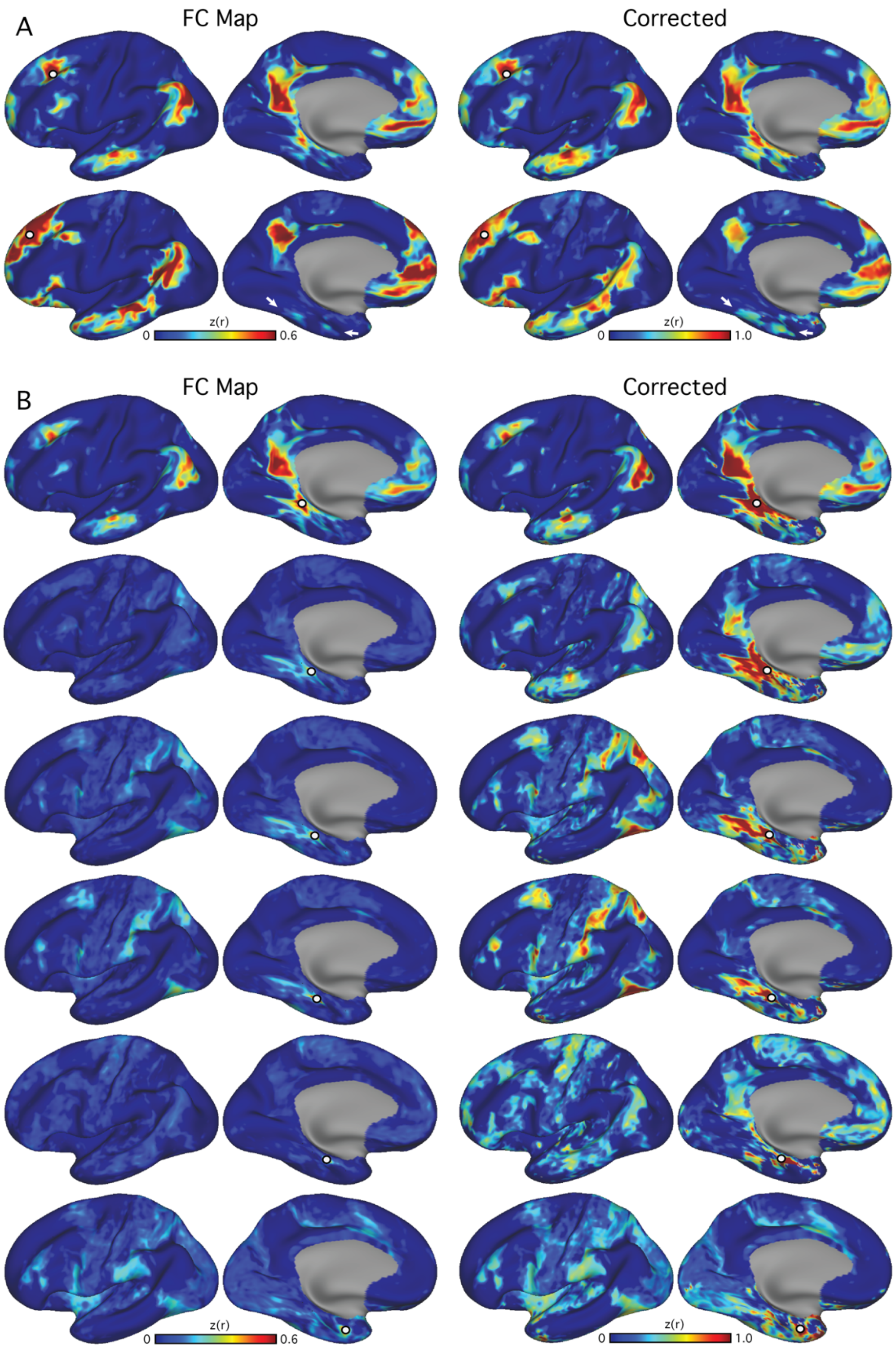
A
FC Map
Corrected
z(r)
0
0.6
z(r)
0
1.0
B
FC Map
Corrected
z(r)
0
0.6
z(r)
0
1.0

Figure S2: Additional Network Representations in Medial Temporal Lobe (MTL) Revealed by Attenuation Correction. Related to Figures 1 and 3 in the main text. The left column shows the functional connectivity maps from subject 4, normalized and averaged across all 24 scan runs, with the visualization threshold dropped to zero so that regions of low correlation strength can be observed. The right column shows the same maps after attenuation correction. A) The unthresholded connectivity maps for Default Network A (DN-A; top row) and Default Network B (DN-B; lower row) are shown, based on prefrontal seed regions. Two possible representations of DN-B are evident in the inferior temporal lobe (ITL; white arrows) at low thresholds (left column), and these representations display increased correlation values when attenuation correction is applied (right column). B) Maps display correlation for a series of seed regions moved incrementally along the MTL towards the temporal pole. Although the functional connectivity maps showed weak correlations with the rest of the cortical surface (left column), reliability correction reveals that a subregion of MTL is correlated with extrastriate visual regions possibly at or near the canonical dorsal attention network (right column). The top two rows show seed regions in parahippocampal cortex that were correlated to DN-A. The remaining rows appear to contain a mixture of signals that are equivocal as to their organization.

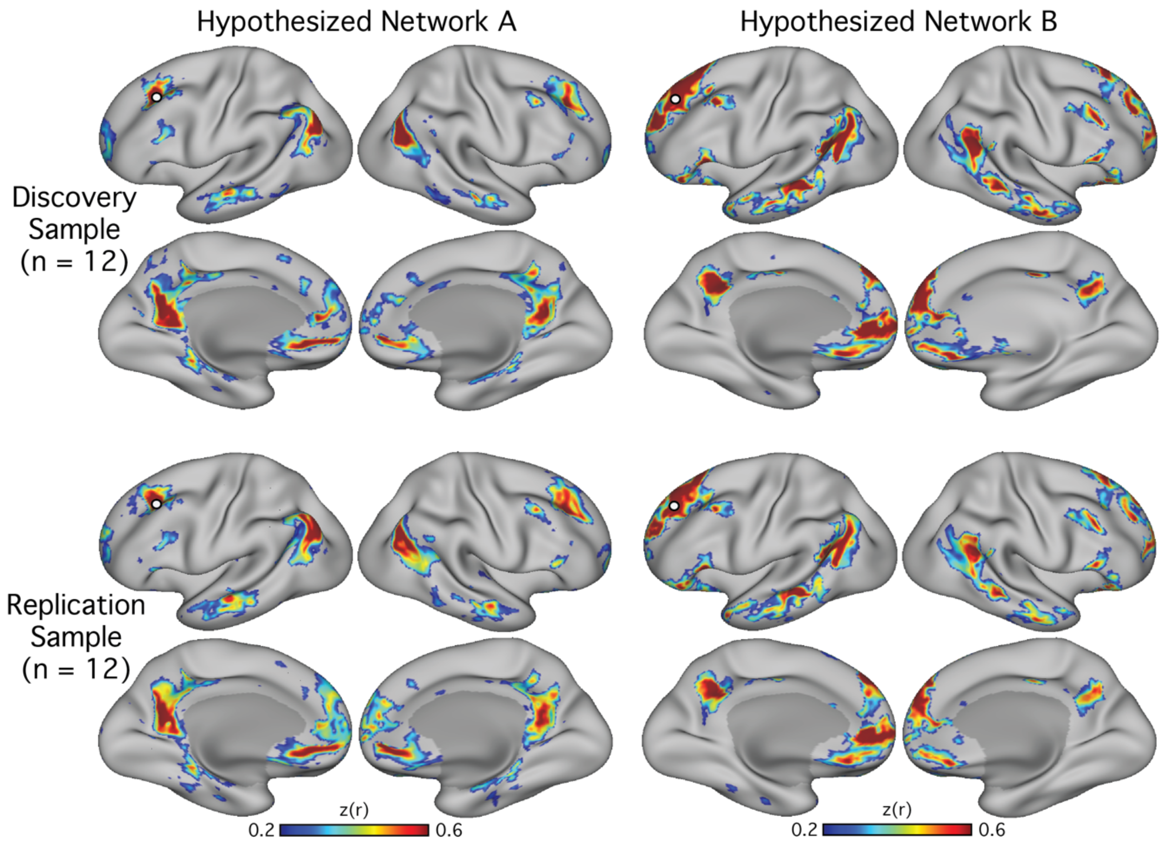

Figure S3: Spatial Details of Network Organization Replicate within the Individual. Related to Figure 1 in the main text. Illustrating the spatial stability of network organization within an individual (S1), functional connectivity maps are shown for the discovery sample (n=12 sessions, top) and the replication sample (n=12, bottom). The columns to the left represent Network A and the columns to the right represent Network B. The prefrontal seed regions are illustrated by white filled circles. In several zones, the networks are interdigitated, including the lateral temporal component of Network B which is surrounded by a component of Network A, and the medial prefrontal network components across multiple regions.

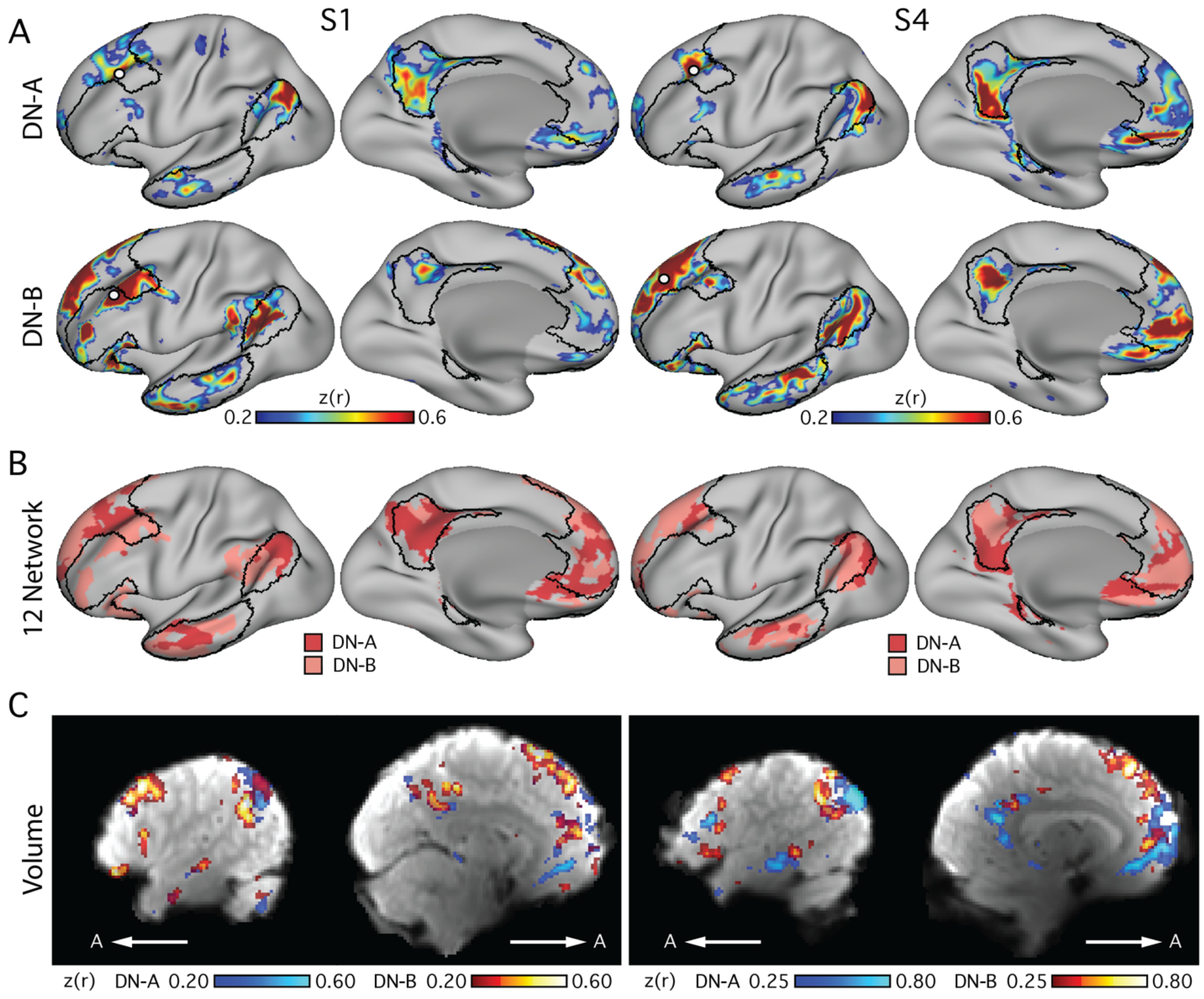

Figure S4: Parallel Interdigitated Networks Are Confirmed Using Multiple Analysis Techniques. Related to Figure 1 and 3 in the main text. Additional images confirm the observed fractionation of the default network using multiple analysis approaches (both seed-based and clustering) and anatomical visualization formats (both surface and volume). A) Seed-based, vertex-by-vertex correlation of data projected to the cortical surface, produced two connectivity maps which both resembled the canonical default network. These two distinct networks (DN-A and DN-B) are shown for subjects 1 (S1) and 4 (S4). The network border from the group-averaged default network is shown in black (taken from the 7-network parcellation from Yeo et al., 2011) to highlight how the DN-A and DN-B both contain regions that fall at or near the canonical default network. B) Networks defined using data-driven parcellation on the

surface are illustrated for each subject. For each individual, the map represents the concatenated time series from each run of the discovery dataset (n=12) clustered into 12 networks using k-means clustering. Of the 12 networks defined, the two networks at or near the canonical default network are displayed to highlight the correspondence between the clustering- and seed-based network analysis approaches. Similar networks were observed using clustering that were juxtaposed and interdigitated in temporal, parietal, medial prefrontal and lateral prefrontal cortices. C) The DN-A and DN-B networks are displayed in the volume using voxel-based functional connectivity to verify the interdigitation between networks observed on the surface is not a consequence of the cortical sampling procedure. Two separate networks are displayed that share the key features of the surface-defined networks reported in the main analysis (shown in A). The DN-A network is shown in the blue colorscale and the DN-B network is shown in the red-white colorscale, both set to a transparency level of 70%. The two networks can clearly be seen to occupy side-by-side regions throughout the brain, particularly in the temporal, parietal and medial prefrontal cortices in S4. Importantly, the distribution of representations from DN-A and DN-B in the medial prefrontal cortex shows that the two networks occupy interdigitated regions in the volume (see rightmost portions of the figures). Note that the lateral views of the brain volume are flipped to match the orientation of the cortical surfaces, such that the anterior (A and arrows) of the brain is on the left.

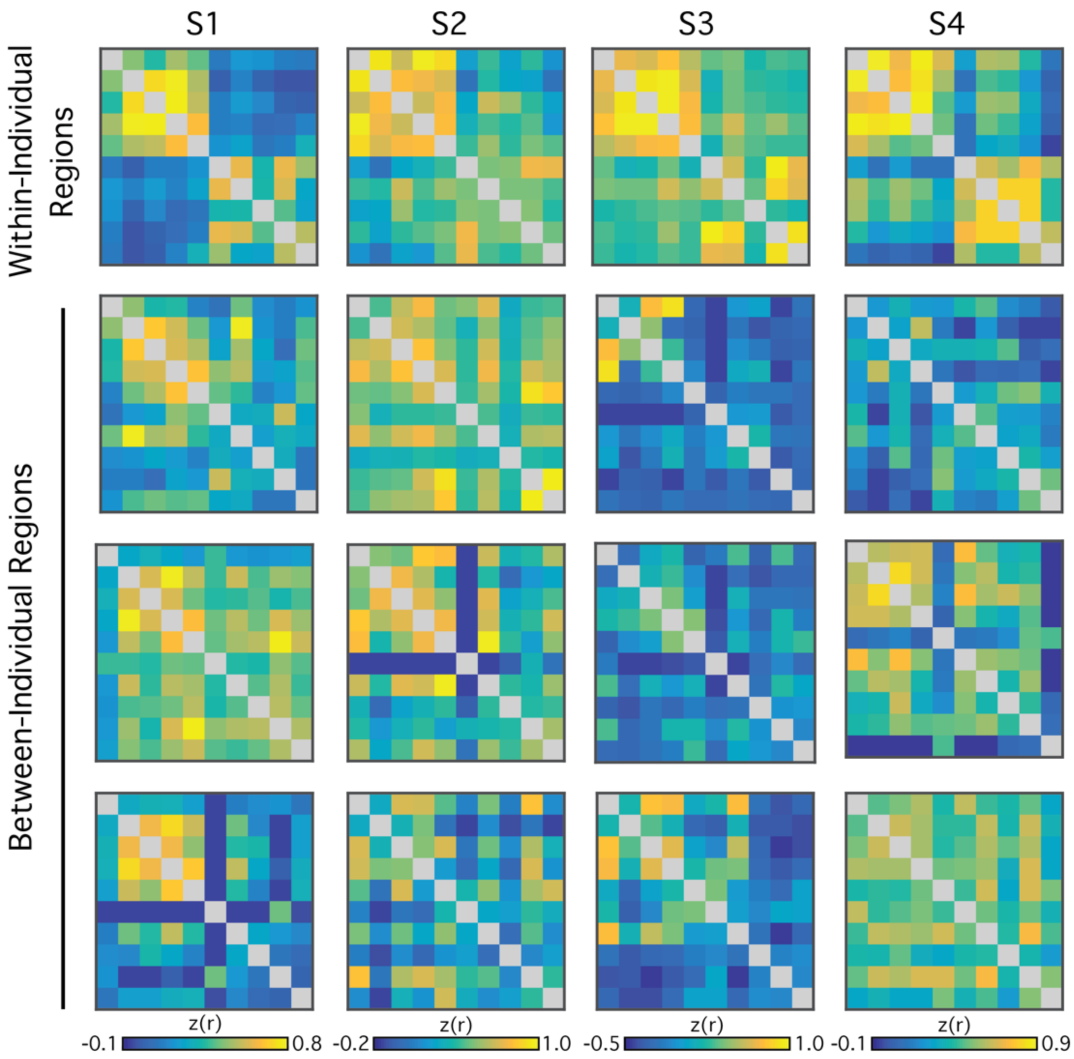

Figure S5: Network Regions Are Not Spatially Aligned Between Individuals. Related to Figure 1 in the main text. Each matrix represents the functional connectivity between the 10 paired regions comprising Default Network A and Default Network B (Figure 1). Values are the mean r-to-z transformed correlations from the 12 samples in the replication dataset. Each column represents data from one subject always using the same scale per subject (shown at the bottom). The top row presents the correlation matrix when the spatial locations of the regions are tailored to that specific subject. In each case, two clusters are evident reflecting the strong within-network correlations of the two dissociated networks (upper left quadrant and lower right quadrant in each matrix). Note further that these clusters of strong positive correlations include region pairs distributed throughout the brain that are often spatially close to regions in

the other cluster. The three rows below each within-individual matrix represent the same original data but with the matrix constructed from the seed region locations defined in the other subjects (e.g., Subject 2's regions applied to Subject 1's data). As can be seen, the clustering patterns break down and the separation of the two networks is absent in many matrices. This result illustrates that the spatial variation between individuals is enough to obscure the clear and reproducible network dissociation that is uncovered within the individuals.

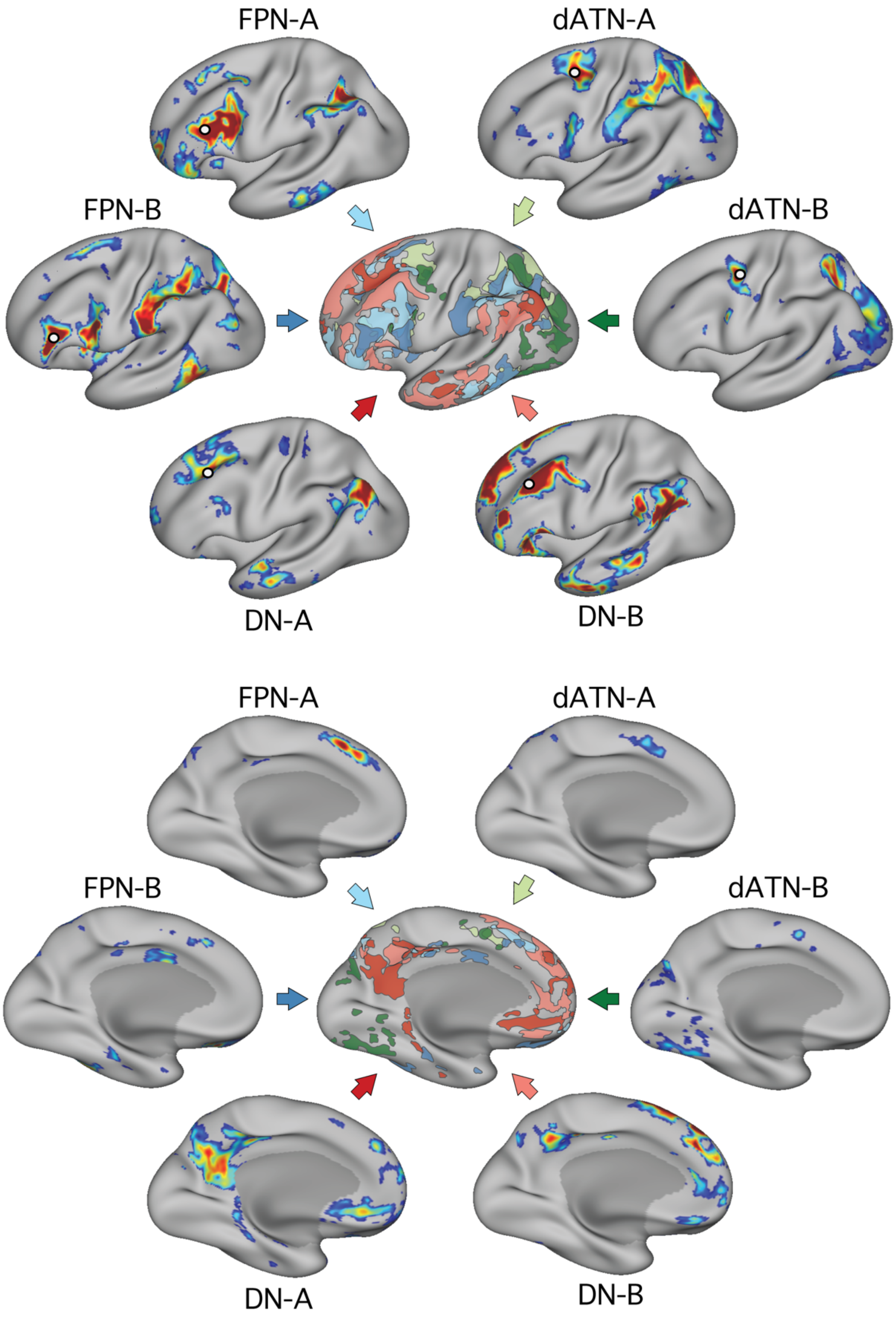

Figure S6: Diagrammatic Representation of Six Parallel Distributed Networks within Another Individual. Related to Figure 8 in the main text. The central figures show an illustration of the

six newly defined networks overlaid on the same cortical surface. The top panel shows the lateral view and the lower panel shows the medial view. The different colors correspond to the canonical network that each network resembles (Red, Default Network, DN-A and DN-B; Blue, Frontoparietal Network, FPN-A and FPN-B; Green, Dorsal Attention Network, dATN-A and dATN-B). The names of the networks are chosen based on prior literature, recognizing that the novel organization identified here may lead to a reconsideration of the functional domains. Data are from Subject 1 (see Figure 8 in the main text for Subject 4).

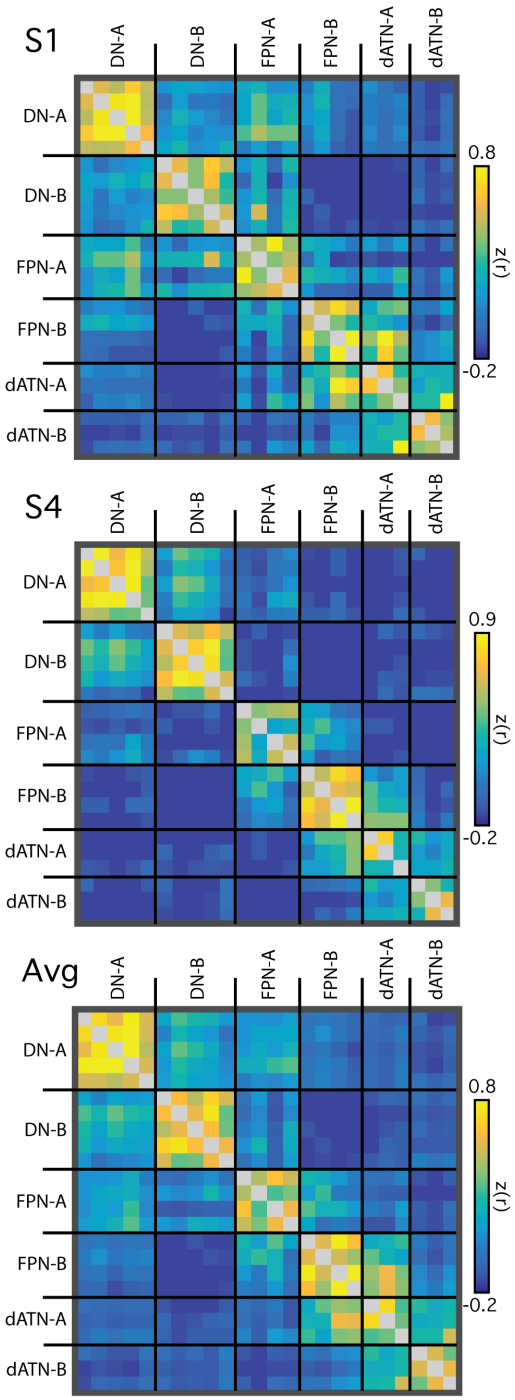

Figure S7: Correlations Between Distributed Networks. Related to Figures 4-8 in the main text. Each matrix illustrates the correlation structure from regions of the six identified networks for subjects S1 (top panel) and S4 (middle panel), and the average of the two subjects (Avg; bottom panel). Region locations are shown in Figure S8. The matrices demonstrate that there may be subtle, differential interactions between networks.

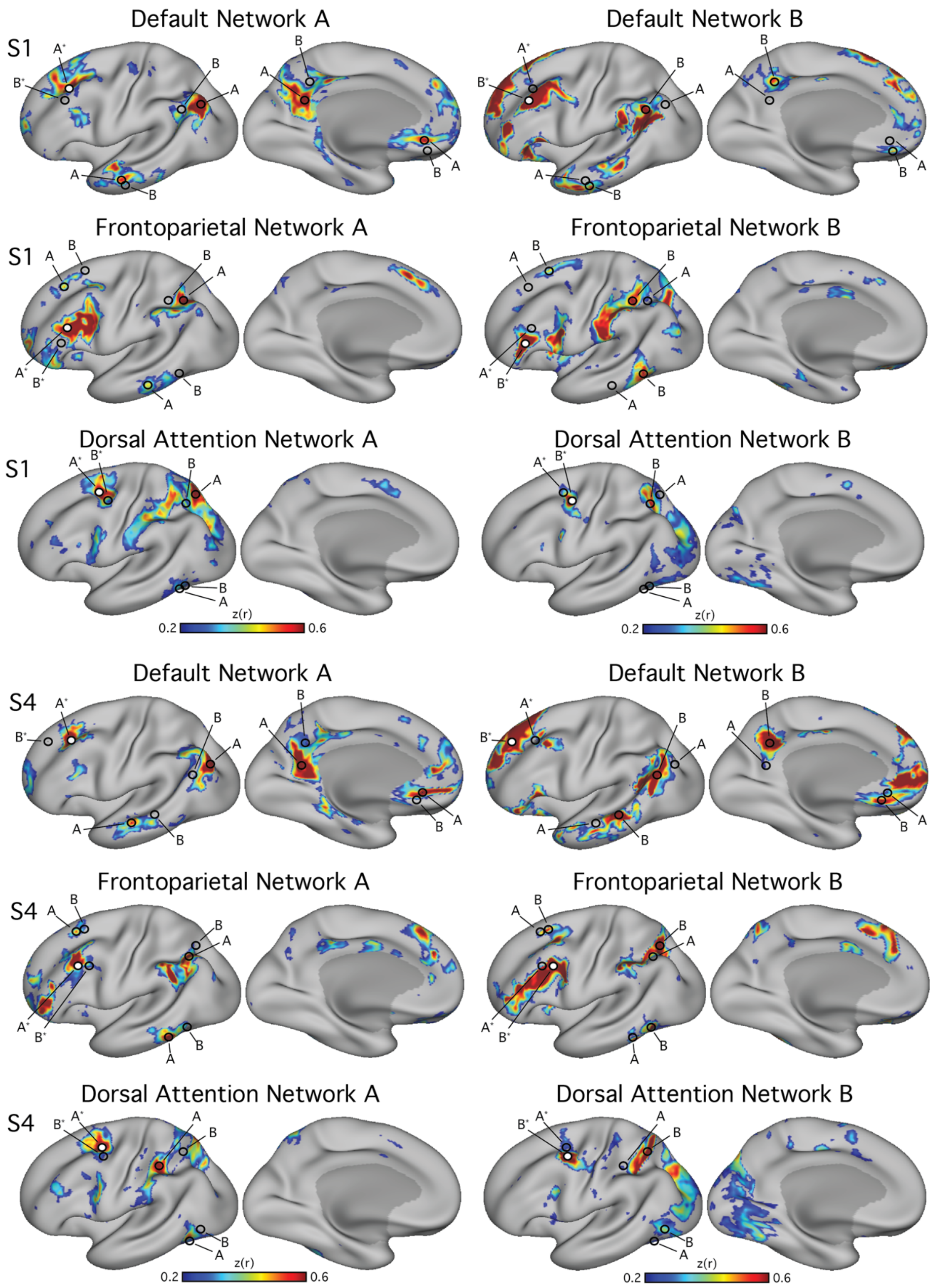

Figure S8: Network Regions for All Six Networks for Subjects 1 and 4. Related to Figures 4-8 in the main text. Panels display the correlation maps for seed regions (white filled circles) placed

in prefrontal cortex to isolate the pairs of fractionated networks within the canonical Default Network (top row for each subject), Frontoparietal Network (middle row), and the Dorsal Attention Network (bottom row). Within each row, the left column shows one network and the right column shows the parallel, spatially distinct network. The labeled regions (hollow circles) were used to estimate network correlations within- and between-networks as presented in Figure S7.
